# Structure-based Molecular Simulations Reveal the Enhancement of Biased Brownian Motions in Single-headed Kinesin

**DOI:** 10.1371/journal.pcbi.1002907

**Published:** 2013-02-14

**Authors:** Ryo Kanada, Takeshi Kuwata, Hiroo Kenzaki, Shoji Takada

**Affiliations:** 1 Department of Biophysics Graduate School of Science, Kyoto University, Kyoto, Japan; 2 Graduate School of Science and Technology, Kobe University, Kobe, Japan; 3 CREST Japan Science and Technology Agency, Kawaguchi, Saitama, Japan; University of Maryland, United States of America

## Abstract

Kinesin is a family of molecular motors that move unidirectionally along microtubules (MT) using ATP hydrolysis free energy. In the family, the conventional two-headed kinesin was experimentally characterized to move unidirectionally through “walking” in a hand-over-hand fashion by coordinated motions of the two heads. Interestingly a single-headed kinesin, a truncated KIF1A, still can generate a biased Brownian movement along MT, as observed by in vitro single molecule experiments. Thus, KIF1A must use a different mechanism from the conventional kinesin to achieve the unidirectional motions. Based on the energy landscape view of proteins, for the first time, we conducted a set of molecular simulations of the truncated KIF1A movements over an ATP hydrolysis cycle and found a mechanism exhibiting and enhancing stochastic forward-biased movements in a similar way to those in experiments. First, simulating stand-alone KIF1A, we did not find any biased movements, while we found that KIF1A with a large friction cargo-analog attached to the C-terminus can generate clearly biased Brownian movements upon an ATP hydrolysis cycle. The linked cargo-analog enhanced the detachment of the KIF1A from MT. Once detached, diffusion of the KIF1A head was restricted around the large cargo which was located in front of the head at the time of detachment, thus generating a forward bias of the diffusion. The cargo plays the role of a diffusional anchor, or cane, in KIF1A “walking.”

## Author Summary

It is one of the major issues in biophysics how molecular motors such as conventional two-headed kinesin convert the chemical energy released at ATP hydrolysis into mechanical work. While most molecular motors move with more than one catalytic domain working in coordinated fashions, there are some motors that can move with only a single catalytic domain, which provides us a possibly simpler case to understand. A single-headed kinesin, KIF1A, with only one catalytic domain, has been characterized by in vitro single-molecule assay to generate a biased Brownian movement along the microtubule. Here, we conducted a set of structure-based coarse-grained molecular simulations for KIF1A system over an ATP hydrolysis cycle for the first time to our knowledge. Without cargo the simulated stand-alone KIF1A could not generate any directional movement, while a large-friction cargo-analog linked to the C-terminus of KIF1A clearly enhanced the forward-biased Brownian movement of KIF1A significantly. Interestingly, the cargo-analog here is not merely load but an important promoter to enable efficient movements of KIF1A.

## Introduction

Time dependent structural information is of central importance to understand detailed mechanisms of biomolecular systems. In particular, biomolecular machines dynamically transit many structurally and chemically distinct states making cycles in state space, by which they fulfill their functions. Unfortunately, no single experimental technique provides sufficient spatio-temporal resolution for them. X-ray crystallography and others provide structural information at high resolution, but this is primarily static. Biochemical and single molecular experiments tell us kinetic and dynamic behaviors, but their spatial resolution is limited. To fill the gap among them, molecular dynamics (MD) simulations have been playing important roles. Yet, due to their size and long time scale involved, atomistic MD cannot cover an entire cycle of molecular machines at the moment [1]. To overcome this limitation, recently, we initiated to use structure-based coarse grained MD (CGMD) methods [2], [3] to mimic the cycle of machines for the case of F_1_-ATPase and others [4], [5]. Notably, most of these machines contain more than one ATPase domains and their coordinated dynamics are crucial to understand the mechanisms [6], [7], [8], [9]. This is an interesting issue, but at the same time, makes the cycle unavoidably complicated. Thus, for the simplicity and clarity, it is good to study those that contain only one ATPase domain and that have much of crystallographic information. In this sense, a single-headed kinesin, KIF1A, is an ideal target system, for which here we performed CGMD simulations mimicking an entire ATP hydrolysis cycle.

Kinesin is a family of molecular motors that move unidirectionally along microtubule (MT) using ATP hydrolysis free energy [10]. In the family, the conventional kinesin, kinesin-1, was experimentally characterized to move toward the plus ends of MT processively with discrete 8-nm steps per one ATP hydrolysis reaction, where the coupling between ATP hydrolysis reactions and 8-nm steps is rather tight [11], . The conventional kinesin is a two-headed motor and has been shown to “walk” in a hand-over-hand fashion by coordinated motions of the two heads [6],[9],[17],[18]. In this sense, it is a surprise that even though KIF1A, a member of kinesin family, is a single-head motor, it still can move processively and directionally along MT, as observed by single molecule experiments [19], [20], [21]. In particular, the mechano-chemical coupling of KIF1A is loose: KIF1A can move back and forth stochastically with an average biased towards the forward direction, with step sizes in multiples of 8-nm. This is in contrast to conventional kinesin that seldom shows backward steps without a large load and that shows a uniform step size of 8-nm per one ATP hydrolysis [19], [20], [21]. Thus, KIF1A must use a different mechanism from the conventional kinesin to achieve the overall unidirectional motions. How KIF1A, with only one head, can generate the unidirectional movements driven by ATP-hydrolysis reaction is unclear in terms of structural dynamics, which we address in this paper by structure-based CGMD. Various molecular simulations have been applied to kinesin as well as other molecular motors [4], [8], [22], [23], [24].

**Figure 1:**
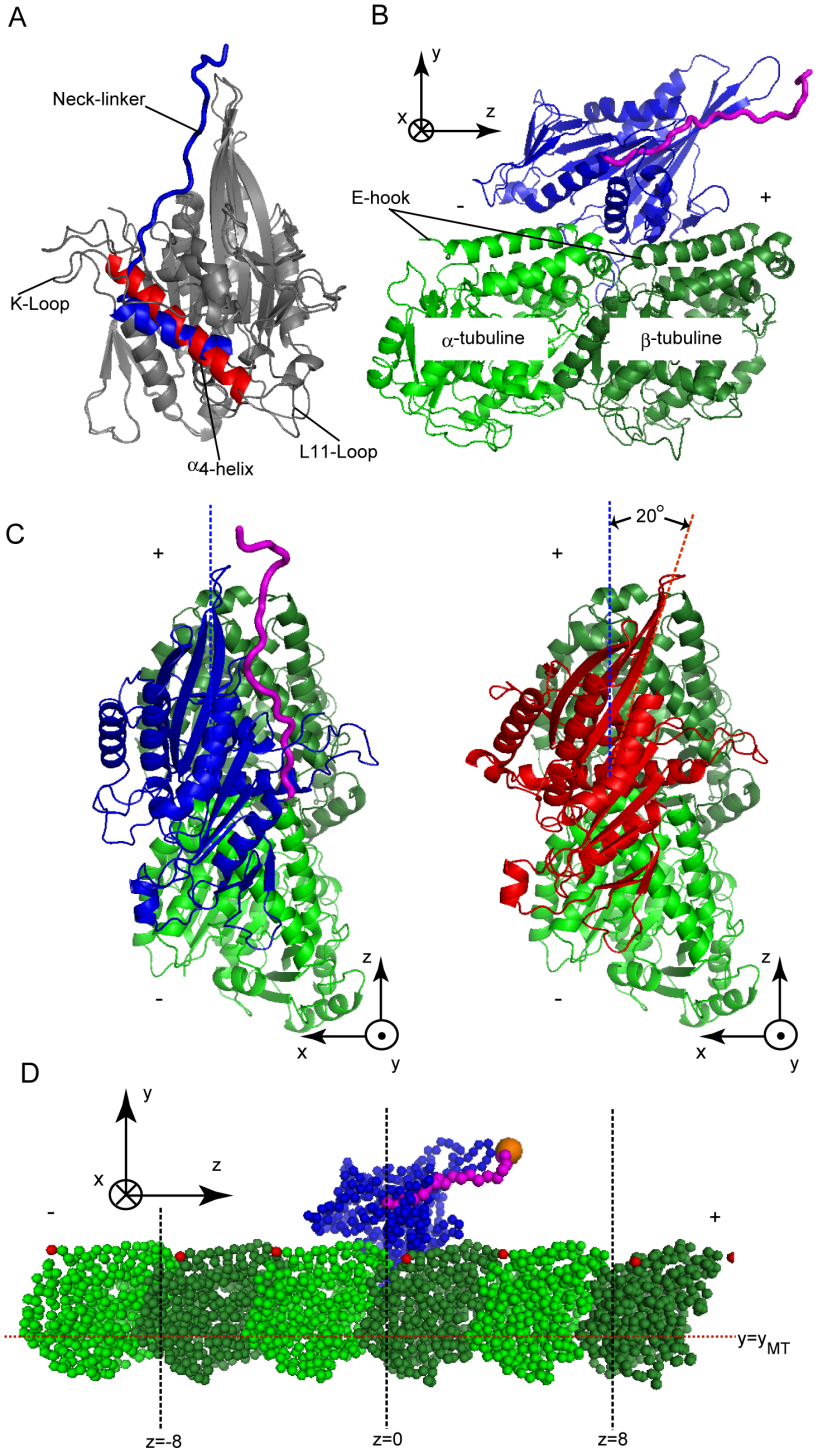
Structure of KIF1A and MT. (A) Superposition for ATP-form and ADP-form structure of the monomer KIF1A. The parts that differ in the two forms are colored: α4-helix for ATP-form in blue and ADP-form in red. (B) The side view of KIF1A (blue and red)-MT (green) complex structure in the ATP-form. The neck-linker is in magenta. (C) The top view of KIF1A-MT complex structure in the ATP-form (left) and the ADP-form (right). (D) The initial structure of the CG simulations. The z-axis is aligned to the long axis direction of MT. The red dots in MT represent E-hooks positions.

For the molecular simulations of KIF1A movements, structural information on nucleotide-dependent conformational change is indispensible. X-ray crystallography provides KIF1A structures in two major conformations; ATP and ADP bound forms [25], [26], [27]. The two forms share the overall fold of the head domain with some changes. One crucial change is in the helix α4; its orientation relative to the rest of the head is rotated by about 20 degrees between the two forms (Fig. 1A, blue for ATP-form and red for ADP-form). Another major change is in the so-called neck-linker region, which is the C-terminus of the head domain: the neck-linker is ordered and tightly docked to the core of the head in the ATP form (magenta in Fig. 1B), while it is disordered and thus invisible in the ADP form. This neck-linker docking/undocking has been implicated as a source of the power-stroke in the kinesin family [28], [29]. The K-loop (L12-loop) and L11-loop, which flank the α4 helix, also show some changes between the two forms (Fig. 1A). Cryo-electron microscopy (cryo-EM) of the KIF1A-MT complex together with X-ray structures of building blocks led to structural models for the KIF1A-MT complex in the two major forms of KIF1A (the ATP and ADP forms) [30], [31]. The modeled complexes show that, in both of the forms, the key interaction sites of KIF1A with MT is the α4 helix, which fits to a groove located between α-tubulin and β-tubulin. In the two forms of KIF1A, the orientation of the α4 helix relative to MT is mostly unchanged, which leads to the 20-degrees rotation of the core domain relative to the long axis (*z*-direction in this article) of MT depending on the bound nucleotide states (Fig. 1C): In the ATP-form, the core adopts the “upright” docking (blue in Fig. 1C left), while in the ADP form the core is rotated about 20 degrees and adopts the “tilted” docking (red in Fig. 1C right) [31]. This core rotation has been suggested to be important for KIF1A movement [26], [31].

The KIF1A-MT complex models provide a clue for the processivity of KIF1A. Regardless of the nucleotide states, the positively-charged K-loop of KIF1A is close to the negatively charged E-hooks, disordered C-terminus regions of α/β-tubluin [26], [31]. Thus, the long-ranged electrostatic attractions between K-loop and E-hooks are assumed to prohibit the KIF1A from completely leaving from MT. This idea is supported by a mutational experiment, in which charge reduction of K-loop decreased the processivity [19].

**Figure 2:**
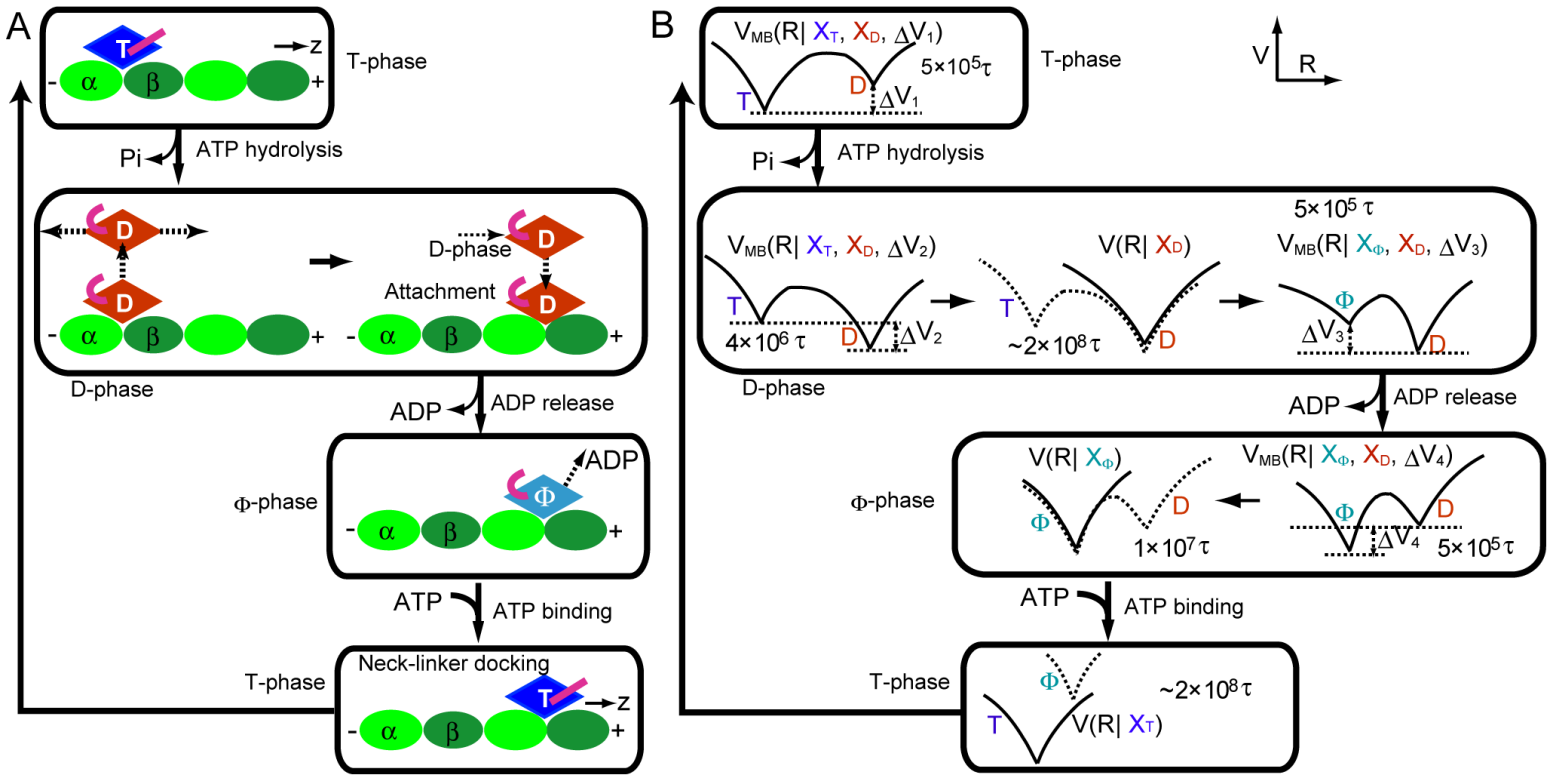
ATP-hydrolysis chemical cycle and CG simulation scheme. (A) The representative model of the ATP hydrolysis cycle of KIF1A. The rhombus with string (magenta) indicates the KIF1A head with the neck-linker. The “T”, “D”, and “Φ” mean ATP-bound, ADP-bound, and nucleotide-free states, respectively. The bent (straight) string means the disordered (docked) neck-linker. The ellipsoids (light green and green) correspond to the αβ-tubulins that compose MT. The entire cycle is divided into 4-phases, T-phase (just before ATP hydrolysis), D-phase, Φ-phase, and T-phase (until neck-linker docking). (B) The CG simulation scheme. The *V*, *R*, and *X* indicate the potential, the temporal conformation, and the reference structure, respectively. The simulation consists of the combination of the two-basin *V*_MB_(R) and single-basin *V*(R) potentials. Each stage is simulated for the indicated time, where the unit of time τ in CG-simulation corresponds to τ∼0.128 ps.

The ATP hydrolysis cycle and its correlation with KIF1A head motion have been investigated previously [19], [20], [21], [26] (see Fig. 2A). The ATP form of the KIF1A head binds strongly to MT (T-phase in Fig. 2A), whereas the direct contact of the ADP-form of KIF1A to MT is weak. Thus, after the ATP hydrolysis and Pi release, the KIF1A head can detach from MT (still loosely bound to MT via electrostatic interactions of the K-loop and E-hooks). The detached head starts diffusion along MT under the constraints generated by the interaction of the K-loop and E-hooks. After the long one-dimensional diffusion along MT, KIF1A can finally find a binding site located at the groove between α- and β-tubulins (D-phase in Fig. 2A). The contact between tubulin and KIF1A induces ADP dissociation from KIF1A leading to the nucleotide free state. In this state, KIF1A binds MT tightly (Φ-phase in Fig. 2A). At the final stage, ATP binding induces neck-linker docking and the rotation of the core (T-phase in Fig. 2A). The above knowledge, however, does not tell us the mechanism of how KIF1A can generate directional movement towards the plus end of MT.

To address the mechanism of directional movement, we designed and conducted a series of molecular simulations employing structure-based CG protein models. The structure-based CG protein models have proven to be useful to study mechanical aspects of kinesin [8], and other molecular motors [4], . Based on the energy landscape view of proteins [32], [33] and structural data for the two forms of KIF1A, we set up single and/or two-basin energy landscapes of KIF1A for every phase of an ATP cycle [34], [35]. Then, the ATP hydrolysis cycle was mimicked by dynamically switching the energy functions of KIF1A in different phases of the cycle (Fig. 2B) [4], [36]. While the full-length KIF1A has a rather long C-terminal tail, we here concentrate on a truncated KIF1A (C351) that was used in in vitro single-molecule assays [19],[20],[21]. We note that, by employing the structure-based CG simulations, our purpose here is not to conduct a single simulation that most accurately approximate the real molecular system, as some parameters in the CG simulations are not accurately derived from atomic interactions. Instead, taking advantage of the speed of the structure-based CG simulations, we systematically conduct a series of simulations for a broad range of these parameter values. These comparative computer experiments are useful for a mechanistic understanding.

## Results

### Computational modeling

We designed a simulation system for one ATP hydrolysis cycle of KIF1A that induces KIF1A motions along MT. The simulation system contains 7 protein subunits: a KIF1A molecule that moves dynamically and three copies of tubulin αβ dimers that were fixed in the form of a segment of single protofilament of MT (Fig. 1D). All the proteins were modeled at a one-bead-per-residue resolution (each amino acid was represented by a bead located at the Cα position).

For KIF1A, we employed structure-based CG models that concisely represent the energy landscape, which is a globally funnel-like shape where the bottom of the funnel can have more than one basin [33]. We focused on a truncated KIF1A C351 (unless otherwise mentioned) since the motility of this type KIF1A is intensively investigated in the single-molecular assay of Okada et al [19], [20], [21]. Conformational changes of KIF1A upon chemical reactions were simulated by the multiple-basin model [35], while the long-time dynamics that do not involve chemical reactions, such as diffusion process, were simulated by a single-basin perfect-funnel (i.e., Go) model [34], [37] (see below and *Materials and Methods* for more details). Protein dynamics was simulated by stochastic differential equation, i.e., the Langevin equation (see below and *Materials and Methods* for more details). The crystal structures of ATP-bound KIF1A (designated as KIF1A(T) hereafter) and ADP-bound KIF1A (KIF1A(D)) are available from the Protein Data Bank and were used in the CG models as reference structures of the corresponding states. For the KIF1A-MT complex structures, the cryo-EM-based models for the ATP- and ADP-bound KIF1A-MT complexes are also available and were used (we designate X_T_ and X_D_ respectively). These models explain the high and low affinities in ATP-bound and ADP-bound forms of KIF1A, respectively, by the number of direct contacts. The structure for nucleotide-free KIF1A (KIF1A(Φ)) is currently unavailable; we assumed that the neck linker is disordered based on experiments, and that the KIF1A(Φ)-MT complex structure X_Φ_ except the neck linker to be the same as that of X_T_ because both states have a similarly high affinity to MT. Using these complexes, we modeled the interactions between KIF1A and MT as a Go-like pair potential (unless otherwise mentioned).

In the current CG model, the interaction strength between KIF1A and MT is a key parameter. First, the interaction strength parameter had to be tuned so that KIF1A(T) can stably bind to MT while KIF1A(D) can detach from MT during the affordable simulation time. This tuning was easy because, as mentioned above, the modeled complex structures of KIF1A-MT have more residue-contacts in the ATP form than in the ADP-form. A more delicate tuning was necessary for the affinity of KIF1A(D) to MT because KIF1A(D) is expected to detach from MT and later reattach. Obviously, a too weak interaction does not lead to attachment of KIF1A to MT, whereas a too strong interaction does not allow the detachment from MT. Via many preliminary runs, we found a certain range of the interaction strength parameters that satisfy these conditions (described in the next subsection).

Our simulation started from the X_T_. KIF1A was bound to the central tubulin αβ dimer (Fig. 1D). We simulated the KIF1A(T) state for 5×10^5^ τ, where τ is the unit of time in CG-simulation, using the multiple-basin potential with two basins: a stable basin at X_T_ and a meta-stable basin at X_D_ structures (Fig. 2B top). The unit of time τ can be mapped to ∼0.128 ps in real time scale based on the diffusion constant of the KIF1A head (see *Materials and Methods* for the detail information). Then, we induced the conformational change to the ADP-bound form by switching the potential so that the X_D_ structure becomes more stable than X_T_ (see the second row and left cartoon of Fig. 2B). With this setting, we simulated the system for 4×10^6^ τ, which is long enough to complete the conformational change to ADP-form. For many samples, KIF1A(D) detached from MT during this period. We note that, throughout the simulations, a constraint potential was applied that represents long-range loose interactions between the K-loop and E-hooks, by which KIF1A cannot move far away from MT (see *Materials and Methods* for details). Then, we conducted a long simulation (2×10^8^τ) with the single-basin Go potential for the X_D_ (the second row and central cartoon in Fig. 2B). The switch from the multiple-basin potential to the single-basin Go potential saves computer time and is done solely for technical reasons. During this period, many trajectories showed KIF1A re-attachment to MT. Once KIF1A attached to MT, we continued the run for another ∼1×10^7^τ and then moved to the next stage. The next stage is a preparation to the subsequent conformational change to the nucleotide-free (Φ) state and uses the multiple-basin model with the stable basin at X_D_ and the meta-stable basin at X_Φ_ for 5×10^5^τ. After that, corresponding to the release of ADP, we induced the conformational change to the nucleotide free form by switching the potential so that the X_Φ_ structure is more stable than X_D_ (the third row right in Fig. 2B). Subsequently, for a long time dynamics, we used the single-basin potential for the Φ state for 1×10^7^τ. Finally, ATP-binding is mimicked by switching the potential to the single potential for X_T_. We simulated the T state for ∼2×10^8^τ, which completes the X_T_ 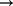
X_D_ 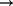
X_Φ_ 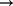
X_T_ cycle.

### CG simulations of stand-alone KIF1A

We now analyze KIF1A movement during one ATP cycle. As in Fig. 2A, it is expected that KIF1A detaches from MT and attaches to MT both in the D-phase. Thus, modeling of the interaction between KIF1A(D) and tubulin is very delicate. Since the CG modeling is unavoidably less accurate, instead of deciding one “correct” interaction strength, we scanned the strength over a certain range.

**Figure 3:**
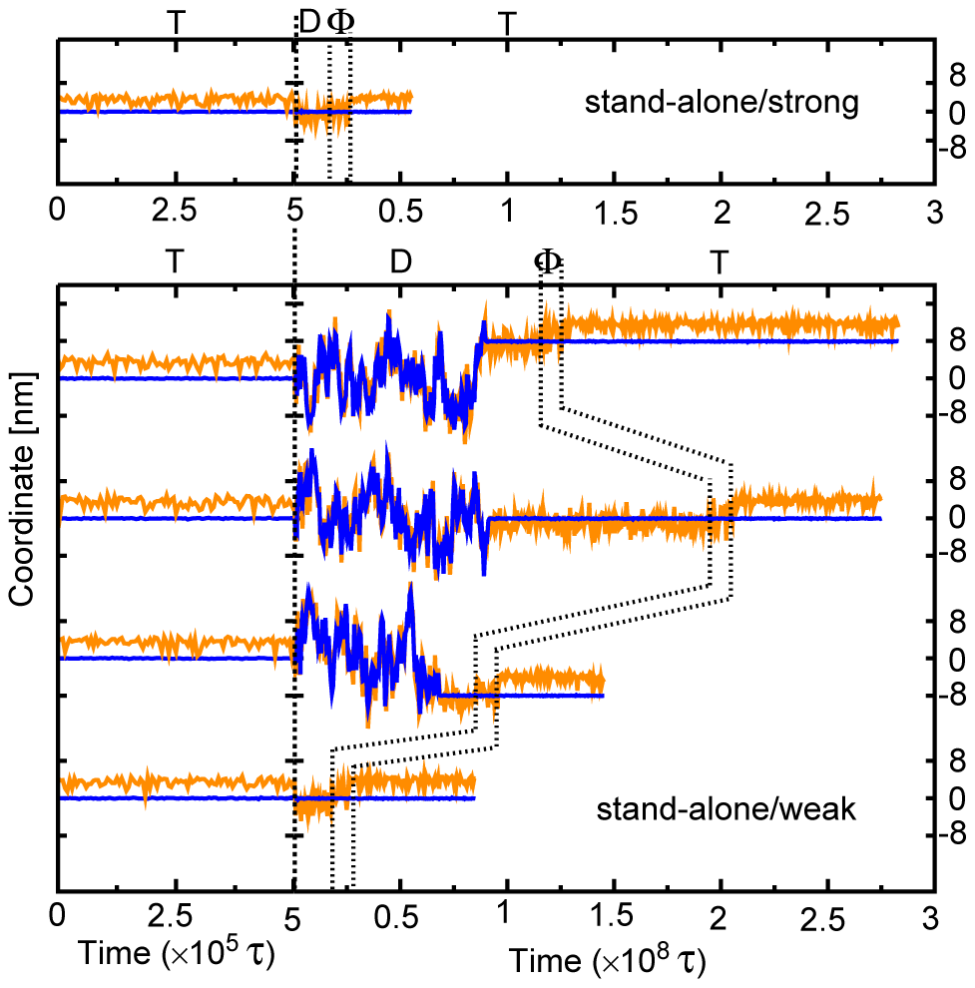
Translational movements of stand-alone KIF1A. The upper (lower) panel shows movements for the case of a strong (weak) interaction between KIF1A and MT denoted as [stand-alone/strong], ε_go_^KIF1A-MT^ = 0.225 ([stand-alone/weak] ε_go_^KIF1A-MT^ = 0.153). The blue and orange lines are z-coordinates of the KIF1A-head (the center of mass) and C-terminal, respectively. Each trajectory contains 4 phases, T, D, Φ, and the next T states split by dashed lines. Note that the scale in x-axis changes at 5×10^5^ τ.

In a strong interaction case (designated as [stand-alone/strong], ε_go_^KIF1A-MT^ = 0.225) (Throughout the paper, the energy unit corresponds to kcal/mol (∼1.7 k_B_T = ∼6.95 pN.nm) although the mapping is rather approximate), we saw KIF1A cannot detach from MT for 99 of 100 trajectories (Fig. 3A) within the simulated time. Whereas, with a weak interaction ([stand-alone/weak], ε_go_^KIF1A-MT^ = 0.153) that was carefully tuned after trial-and-errors, we found that KIF1A can detach from MT and attach to MT (the first three cases in Fig. 3B) for 186 of 235 samples (∼80%). The rest 49 samples did not show detachment (bottom in Fig. 3B). The first, second, and third cases in Fig. 3B illustrate the one forward step (+8 nm), the zero-step (0 nm), and the one backward step (−8 nm) within one ATP hydrolysis cycle, respectively (For an example of stand-alone KIF1A movements for [stand-alone/weak], see Supporting Information Video S1). Of the 186 cases that KIF1A detached from and attached to MT within one ATP chemical cycle (T
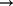
D
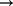
Φ
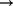
T), the positions of KIF1A at the end of simulations were +8 nm (the forward step) for 44 cases, 0 nm (zero-step) for 92 cases, and −8 nm (the backward step) for 50 cases (For statistics, Table 1). We note that the system contained only 3 pairs of tubulin αβ's that correspond to kinesin binding sites of +8 nm, 0 nm, and −8 nm so that possibilities of two steps were out of the scope here. On average, no significantly biased move was observed. Apparently, this does not explain the in vitro single molecule experiments that found forward biased moves.

**Table 1:** The statics of the translational movements of the KIF1A head in one ATP-hydrolysis cycle.

	#sample	No-detach	+8 nm	0 nm	−8 nm	?^2^ *	P-value**
stand-alone/strong	100	99	0	0	1	1.00	3.17×10^−1^/Accepted
stand-alone/weak	235	49	44	92	50	0.383	5.36×10^−1^/Accepted
Cargo/strong	109	0	52	56	1	49.1	2.46×10^−12^/Rejected
cargo/weak	150	0	43	81	26	4.19	4.07×10^−2^/Rejected
Stand-alone/weak/DH	80	6	12	46	16	0.571	4.50×10^−1^/Accepted

* ) The chi-square test for the null hypothesis “If the KIF1A head succeeds in detaching itself from MT, the forward and backward binding events are equally probable.”

** Based on the null hypothesis, the probability to observe the current data. The null hypothesis was “accepted” or “rejected” by the significance level 0.05. Data here do not include re-detachment and re-attachment processes, which did not produce any significant bias (see Fig. 9 and sub-section “The statics of the re-detachment and the reattachment process”).

The simulations above did not consider electrostatic interactions at all, which may have affected the results. Indeed, recent work by Grant et al reported forward bias of two-headed kinesin landing due to electrostatic interactions [22]. We thus added the electrostatic interactions between KIF1A and MT by the Debye-Huckel formula and repeated the same set of simulations for 80 samples for the case of [stand-alone/weak/DH]. We set the salt concentration of 50 mM, and put +1 charges to all the Lys, Arg, and His residues and −1 charges to all the Asp and Glu residues in the simulated system. Of 80, 6 samples did not show detachment, 12 samples showed one forward-step (8 nm), 16 samples showed one backward step, and 46 samples returned to the original site (see Fig. S1). Thus, inclusion of the simple electrostatic interactions did not produce forward-biased movements although it changed the trajectories to some extents (see Figs S2, S3, S4, S5, S6). We further tried simulations with many different sets of parameters never finding biased motions.

Our results is apparently inconsistent with the biased binding mechanism proposed in [21]. There can be two possibilities. 1) Some fine effect which is not included in our CG simulations, such as more accurate electrostatic treatment by Grant et al, is responsible for the forward biased binding. 2) The forward-biased binding is not realized. Further work is necessary to solve the issue.

### KIF1A linked to a large cargo-analog

In struggling for search of models/situations that exhibit the forward biased move of KIF1A, we came up with a situation that a large cargo-analog is attached to the C-terminus of the neck-linker of KIF1A. The cargo-analog is modeled as a large sphere of ∼1 µm radius, and thus has very small diffusion constant. There are some in vitro experiments for myosin, as well as another kinesin mutant, that suggest the importance of diffusion anchor linked at the end of motor proteins for processive and directional movements [38], [39]. Technically, we added a mass point with large friction coefficient to the C-terminus of the neck linker.

With a large cargo-analog, we first used a strong interaction between KIF1A and MT ([cargo/strong], ε_go_^KIF1A-MT^ = 0.225, the same strength as the case of [stand-alone/strong]), and simulated one ATP cycle for 109 samples. We modeled the cargo as a sphere of radius 3000 times as large as the radius of an amino acid, which is ∼1 µm. Assuming the same density as amino acids, the mass of the cargo scales as 3000^3^ times as large as that of an amino acid. The Stokes-Einstein law *D* = k_B_T/6πηr, where η is water viscosity: ∼0.8 m [Pa s] and r is the radius of the particle, gives that the diffusion constants *D*_cargo_ for the cargo is 3000 times smaller than the diffusion constant of an amino acid. (See *Materials and Methods* for the detailed information).

**Figure 4:**
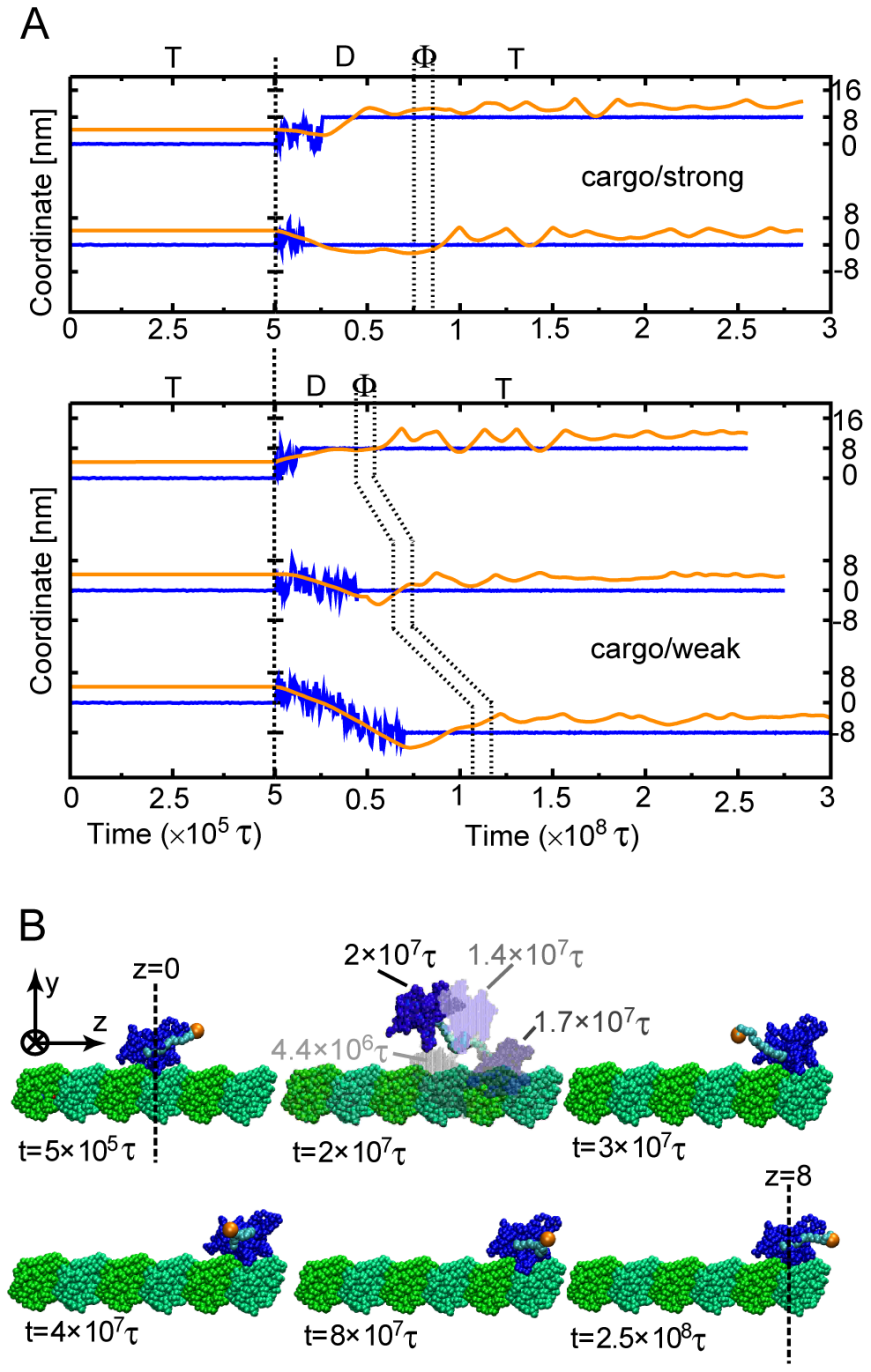
Translational movement of KIF1A linked to a large cargo and some snapshots. (A) The upper (lower) panel shows movements for the case of a strong (weak) interactions between KIF1A and MT denoted as [cargo/strong], ε_go_^KIF1A-MT^ = 0.225 ([cargo/weak] ε_go_^KIF1A-MT^ = 0.153). The blue and orange lines are z-coordinates of the KIF1A-head (the center of mass) and C-terminal, respectively. Each trajectory contains 4 phases, T, D, Φ, and the next T states split by dashed lines. (B) Some snapshots in the first case (8-nm forward step) in [cargo/strong] case. The second frame draws 4 snapshots at 4.4×10^6^ τ, 1.4×10^7^τ, and 1.7×10^7^τ, and 2×10^7^ τ. The simulation treated the default-cargo as ∼1 µm-sized sphere, but the snapshots used much smaller ball to “visualize” the cargo position.

**Figure 5:**
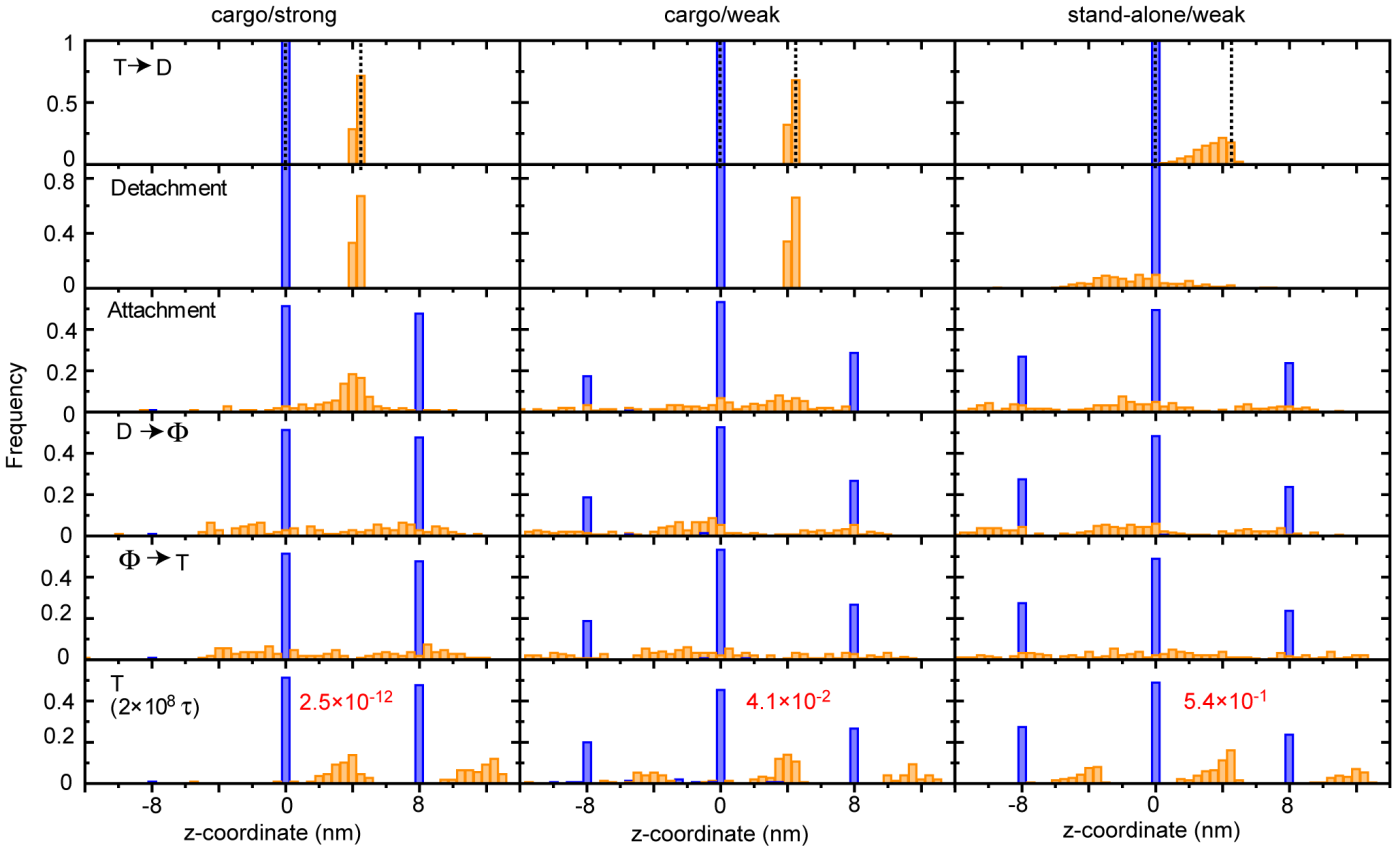
Position distribution of the KIF1A-head and C-terminus in one ATP-hydrolysis cycle. The left, middle, and right panels show the position distributions of KIF1A-head (blue) and C-terminus (orange) for [cargo/strong], [cargo/weak], and [stand-alone/weak] cases, respectively. One ATP cycle was split into 6-phases. In the top panel, the vertical dashed lines show the initial positions (*z* ∼4.25 nm). In the bottom panel, the red-letter values indicate the P-values (see Table 1 for more detail).

In the simulations, we found most samples either moved one-step forward (52 of 109 cases, an example in the upper panel of Fig. 4A top and Video S2) or re-bound to the original site (56 of 109 cases, the upper panel of Fig. 4A bottom), while almost no case of the backward step was found (Table 1). In ATP-bound state (t<5
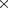
10^5^τ), KIF1A head kept binding to MT firmly and the cargo-analog did not move significantly at 4 nm in front of the head corresponding to the length of the neck-linker (a snapshot in Fig. 4B top, a histogram in Fig. 5 left). Immediately after the ATP hydrolysis, KIF1A head detached from MT quickly. After the detachment, KIF1A head exhibited quasi-one dimensional diffusion along MT, while, due to the large friction, the cargo-analog did not move significantly. Thus, the fluctuation of the KIF1A head was restricted around the almost-fixed cargo located 4 nm in front (Fig. 4B and Fig. 5 left). The cargo-analog played a role of an anchor (or a cane). After some diffusion, the detached head finally re-bound to MT. Because of the limited range of diffusion, the re-binding site was either the forward site (+8 nm) or the original site (0 nm). After the attachment on MT, we changed the state of the system from ADP-state to Φ-state, which did not lead to any marked difference in the movement of the cargo or the head. After that, ATP binding to KIF1A induced the neck-linker docking that moved the position of the cargo-analog, which is about 8 nm in case of the forward step (Fig. 5 left). Thus, after one ATP cycle (T
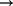
D
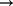
Φ
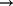
T), the 8-nm or 0-nm displacements of the cargo-analog as well as the head were realized stochastically.

With a weak interaction between KIF1A and MT ([cargo/weak], ε_go_^KIF1A-MT^ = 0.153), we still found clear forward bias (the bottom panel of Fig. 4A) although the details were different. In particular, due to a weaker interaction, the average time for the head diffusion increased, which resulted in larger exploration by one-dimensional diffusion and appearance of the one backward step (26 of 150 samples) as well as the one forward (43 of 150) step, and the zero step (81 of 150) (Fig. 5 middle). As noted before, our simulation system included only the three binding sites and thus two forward or backward steps were not realized by design. For comparison, Fig. 5 right shows the histogram of the move for the case of [stand-alone/weak], confirming that no significant bias is observed.

### The detachment process

We now focus on the detachment process of the KIF1A head from MT after the ATP hydrolysis and Pi release. Upon the potential switch from ATP- to ADP-state at t = 5
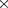
10^5^ τ (Fig. 2B top to the second row left), the decrease in the number of residue-contacts between KIF1A head and MT led to the reduced binding energy, which could induce the detachment of KIF1A head.

**Figure 6:**
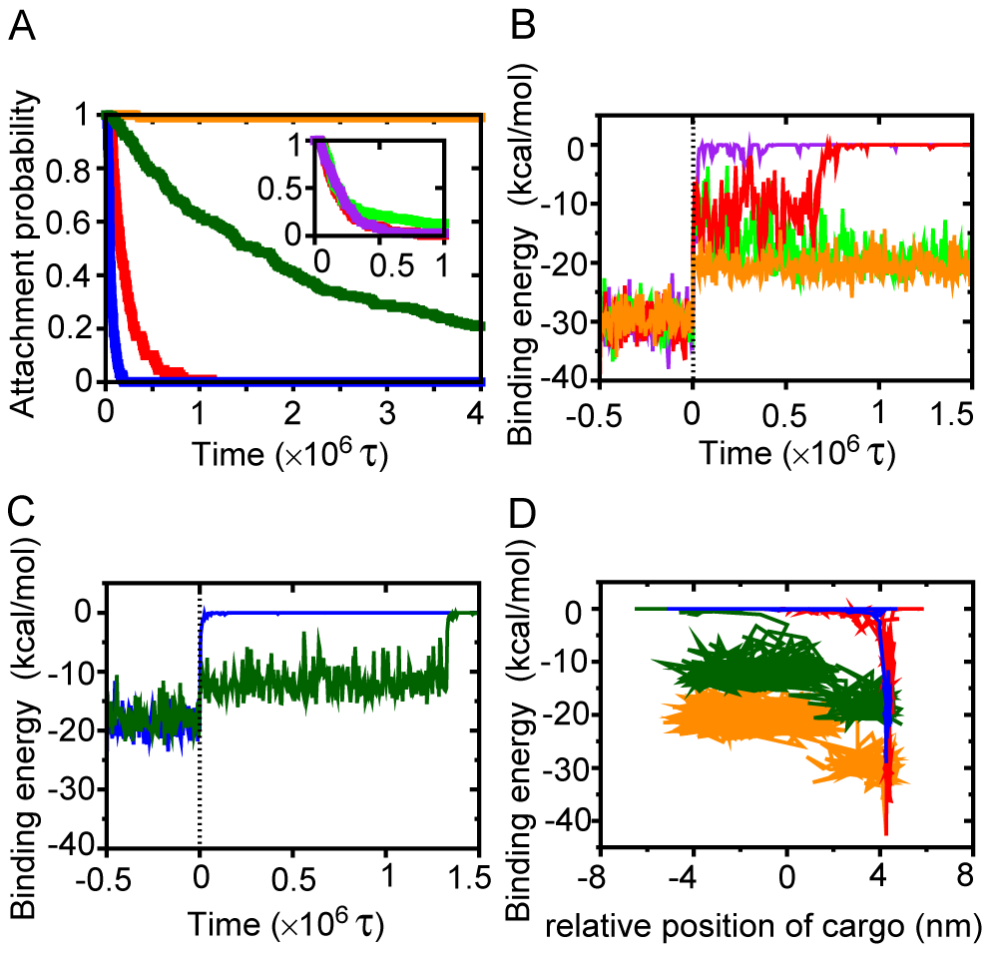
Representative time courses of dissociation of the head from MT after T → D switch. (A) The probability of the KIF1A head still attached on MT as a function of duration time from the T → D switch. The red, blue, orange, and green lines correspond to the cases of [cargo/strong], [cargo/weak], [stand-alone/strong], and [stand-alone/weak], respectively. The inset is the corresponding results of [cargo/strong] with the three sizes of cargoes, a smaller cargo with friction 2.5×10^−8^ (light-green), a middle one with 1.1×10^−8^ (red), and a larger one with 0.53×10^−8^ (magenta). (B)(C) The binding energy between the KIF1A head and MT as a function of duration time from the T → D switch for case of the strong interaction (B), and for the case of the weak interaction (C). (D) A dissociation trajectory in the plane (E_B_, z_relative_) after the T → D switch, where E_B_ is the binding energy between KIF1A and MT, and z_relative_ is the position of the cargo-analog (the C-terminal) relative to that of the head (i.e., z_cargo_-z_head_). The color assignments in (B) (C) (D) are the same as those in (A).

Interestingly, with the strong interaction, the stand-alone KIF1A simulation showed the KIF1A head detachment with the probability 1%, whilst the simulation with the cargo-analog promptly induced the head detachment with the probability 100% (Fig. 6A). Thus, clearly, the cargo-analog enhanced the KIF1A head detachment from MT. Even with the weak interaction between KIF1A and MT, the detachment probability was 79% for the stand-alone KIF1A (Fig. 6A).

With the strong interaction, we tested the detachment process with three cargo sizes (and thus three frictions and masses) (the inset in Fig. 6A); the small (light-green), the middle-size (red, the default one) and the large (purple) cargoes correspond to the radii of 2000, 3000, and 4000 times of one amino acid, respectively. Technically, for given radii, masses and frictions were scaled according to Stokes-Einstein law in the same way as described. We see that KIF1A did not detach from MT with the probability 11% for the case of the small cargo, while the detachments probabilities were 100% for the system with the middle or the large cargo. Thus, the relatively large friction/mass cargo promoted the detachment of the KIF1A head.

In the complex of KIF1A-MT, the α4 helix of KIF1A fits into a groove of MT. When the ATP hydrolysis occurs in the KIF1A head bound to MT, the head tends to make conformational change from ATP-form to ADP-form. With the constraint on the α4 helix, the conformational change would induce about 20 degree clockwise rotation of the head relative to the microtubule (viewed from the top as shown in Fig. 1C left to right), which increases the distance between C-terminal of the head and the cargo rapidly. Then, a tag-of-war between the head and the cargo takes place. When the cargo is sufficiently large, the cargo is less mobile and wins the tag-of-war, thus finally pulling the KIF1A head out of MT.

Fig. 6B illustrates time series of the binding energy for the strong interaction case. For the case of [stand-alone/strong] (orange), the binding energy was weakened from ∼−30 kcal/mol in T-state to ∼-20 kcal/mol in D-state, but the latter was strong enough to hold the KIF1A head stably. For the large cargo case (purple), upon ATP hydrolysis, KIF1A promptly detached from MT. For the cases of small (light-green) and the middle-size (red) cargoes, T
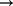
D switch immediately weakened the binding energy to ∼−12.5 kcal/mol, which were followed either by detachment or by the relaxing to the binding energy ∼−20 kcal/mol in D-state (light-green). This transient intermediate state with the binding energy ∼−12.5 kcal/mol corresponds to the frustration imposed by the immobile cargo. Similar behavior was seen in the case of the weak interaction (Fig. 6C).

We found it interesting to plot the trajectories in the plane (z_relative_,E_B_) [z_relative_: the relative position of the cargo (z_cargo_-z_head_), E_B_: the binding-energy] both for the cases with and without the cargo-analog (Fig. 6D). Trajectories start from the right-lower area in (E_B_, z_relative_) plane. With the large cargo (red and blue), after the relaxation of the binding energy from the initial condition to 0 k_B_T (the detachment), the cargo-analog moved. Whereas, without the cargo-analog, the C-terminus fluctuation occurred first and then KIF1A head may or may not detach from MT (orange and dark-green). The difference comes from the different time scale for the mobility of the cargo-analog.

Experimentally, the binding free energy of KIF1A head with MT was estimated from the dissociation constant as ∼−20 k_B_T in the ADP bound state [19]. In the current simulations, the binding energies in the D-phase are −35 k_B_T for the strong interaction case (see Fig. 6B) and −17 k_B_T for the weak interaction case (Fig. 6C). Note that the experimental estimate is the free energy about the standard state, while the estimates from simulations are merely interaction energies. Thus these numbers should not be quantitatively compared. With the uncertainty in mind, perhaps, the real binding strength may fall in between the strong and the weak interaction cases.

### The diffusion and the attachment

**Figure 7:**
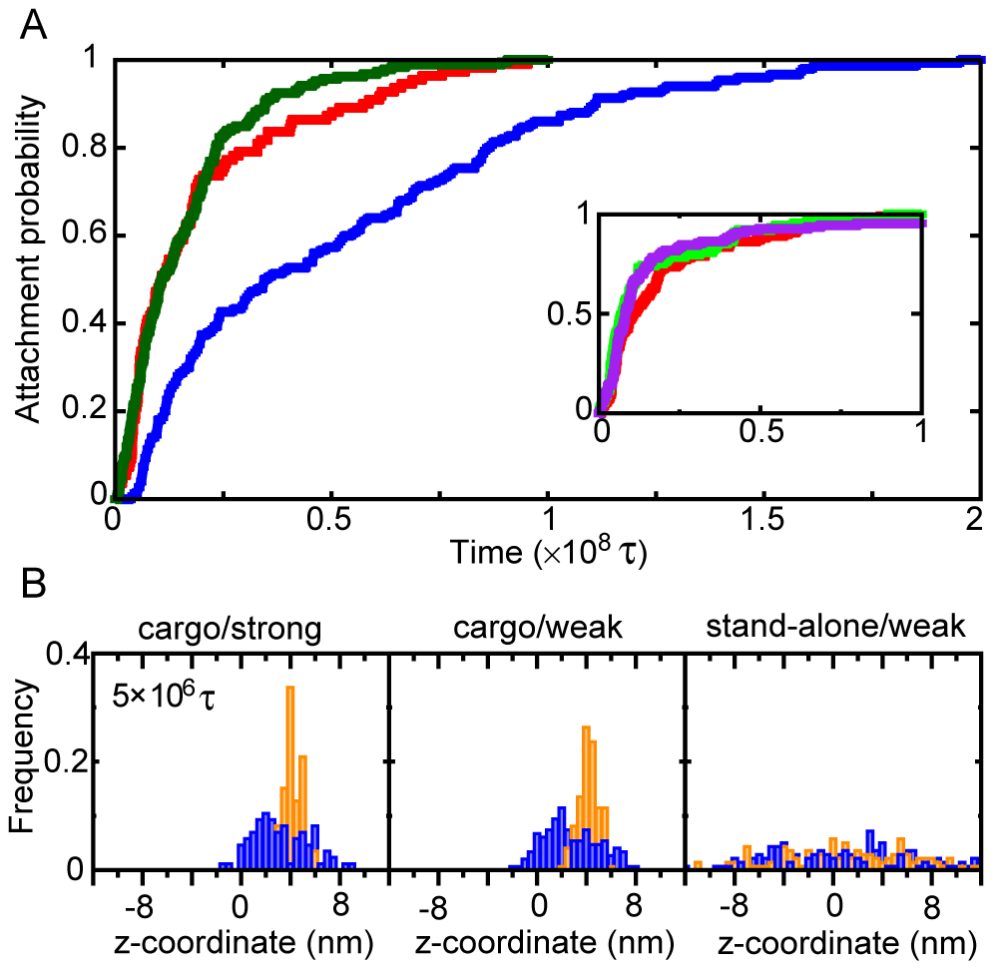
The attachment and the diffusion processes in D state. (A) The attachment probabilities as a function of the duration time after the dissociation of the KIF1A head from MT for the cases of [cargo/strong] (red), [cargo/weak] (blue), and [stand-alone/weak] (green). The inset is the corresponding results of [cargo/strong] with the three sizes of cargoes (See Fig. 6 captions for the color assignments and the cargo-analog sizes) (B) The statistics of the head and the cargo-analog positions at 5×10^6^ τ after the dissociation of the head from MT in D-state. The left, middle, and right panels correspond to the case of [cargo/strong] [cargo/weak], and [stand-alone/weak]. The meanings of the blue and orange bar are the same as those in Fig. 5.

Next, we analyze the diffusion and the attachment processes of KIF1A head after the detachment in ADP-state (Fig. 7). The attachment rate for [cargo/strong] is larger than that for [cargo/weak], as expected. Interestingly, the attachment rate for [cargo/weak] was much smaller than that for [stand-alone/weak], probably due to the restricted motions anchored by the large cargo-analog. Thus, the large cargo-analog enhanced the detachment, but retarded the attachment.

Fig. 7B shows a transient histogram for the *z*-coordinates of the KIF1A head and of the cargo-analog soon after the detachment from MT. With the cargo-analog (Fig. 7B left and middle), its positions were nearly fixed, whereas the head fluctuated broadly (∼4 nm in both directions), which coincides with the length of neck linker. We note that, since we measure the diffusion after the detachment from MT, the histograms for [cargo/strong] and for [cargo/weak] are nearly the same: The diffusion process itself (up to the attachment) was not affected by the interaction strength. As the diffusion time increases, the cargo-analog slowly moves, which enables the head to reach the backward site, as well as the forward site. For the stand-alone case (Fig. 7B right), C-terminus position diffused quickly after the detachment from MT, and the distributions of the C-terminus and the head were nearly symmetric about the starting point (0 nm).

In the simulations, the average times τ_attachment_ for attachment of the KIF1A head to MT for the system cargo/strong and cargo/weak were ∼0.2×10^8^ τ (∼2.5 µs) and ∼0.5×10^8^ τ (∼6.4 µs), respectively. A rough estimate of the diffusion length in this time scale is 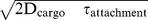
∼1.2–1.9 nm, which is small.

### Neck-linker docking after ATP-binding

**Figure 8:**
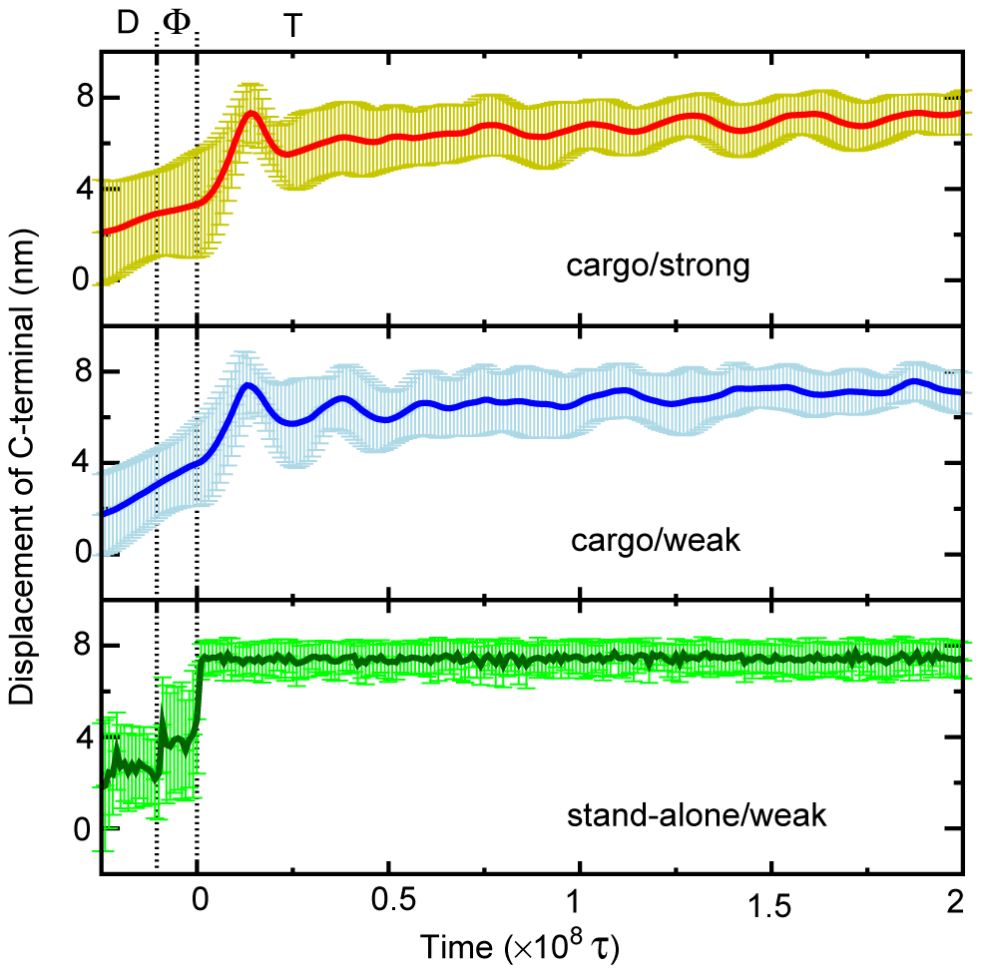
The cargo displacement upon the neck-linker docking in T-state. The time evolutions of the cargo-analog (C-terminus) as a function of time for the cases of [cargo/strong] (the upper), [cargo/weak] (the central), and [stand-alone/weak] (the lower). The time zero corresponds to the time of the switch from Φ to T state corresponding to the ATP binding. The statistics includes only samples that landed at the 8-nm forward binding site.

After ATP binding, the neck-linker docked to the head core. The neck-linker docking moves the cargo-analog by about +8 nm when the head landed to the forward site (Fig. 8). The docking rate depends on the size of the cargo-analog, as expected.

### The statics of the re-detachment and the re-attachment process

**Figure 9:**
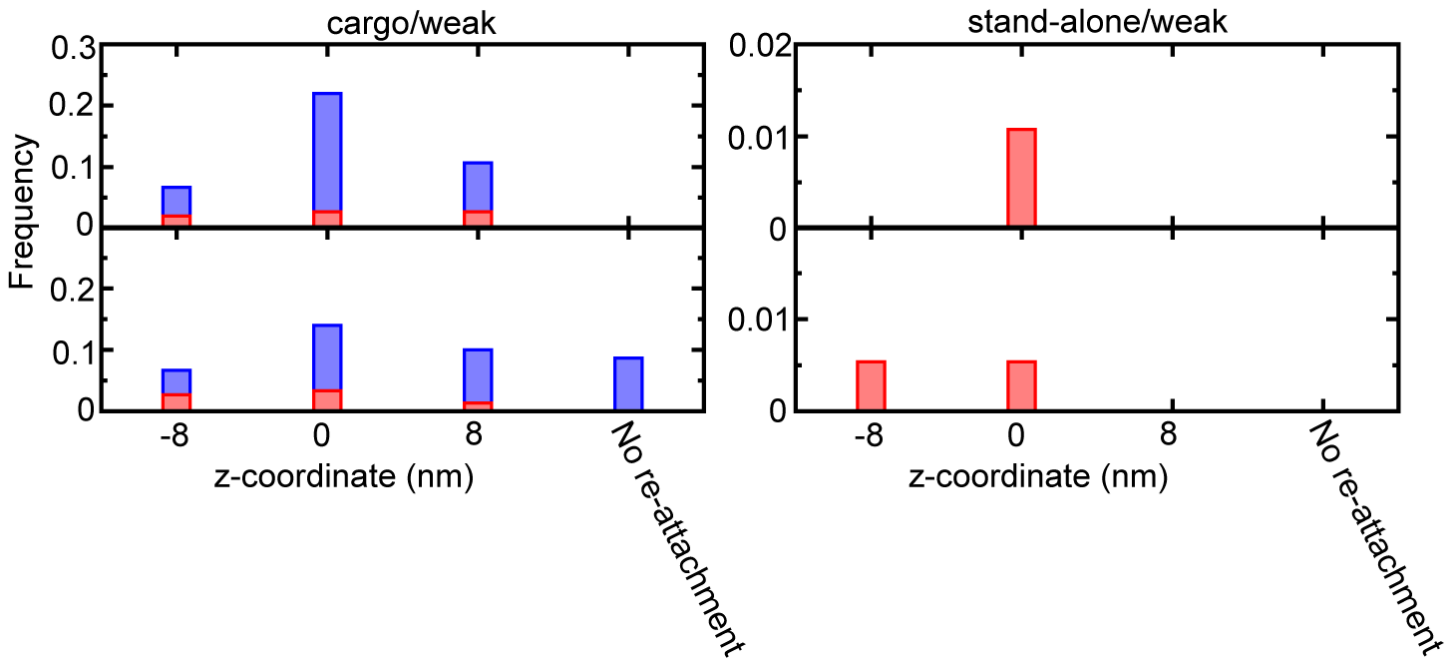
The statics of the re-dissociation and the reattachment process. The upper panel shows the re-dissociation probability at each-binding site, while the lower one indicates the reattachment probability of the head. The left one and the right one are for the cases of [cargo/weak] and [stand-alone/weak], respectively. The state that caused the re-dissociation/reattachment event was shown by different colors; the red (D-state), and blue (T-state).

Only in the cases of the weak interaction, after the attachment of the head onto MT, occasionally the head re-detached from and then re-attached to MT (Fig. 9). This extra processes, being not coupled with ATP cycle, did not produce significant bias in the KIF1A move.

### The stepping statics of KIF1A with the various initial position of the cargo-analog

In the above simulations, the cargo was always placed at z∼4.25 nm based on the ATP-form reference structure, which may raise a concern that this specific initial positioning may affect the stepping statics. To address this concern, we performed the same type of one-ATP cycle simulations with various initial cargo positions z; z = 4.75, 4.25, 3.75, 3.25, 2.75, 2.25, 1.75, and 1.25 nm especially for the cargo/strong case. This range corresponds to the range of cargo found at the end of original simulations (see Fig. S7 A the upper panel which shows the distribution of the probability density for the relative position of the cargo at the end of original simulation). From each of these cargo (initial) positions, 10 simulations were conducted. From the initial position of the cargo: z>3 nm, we found clear forward-biased moves, whereas z<3 nm, the head seldom detached from MT (the lower panel of Fig. S7A). Overall, by distributing the initial cargo positions, the forward bias is somewhat reduced on average. Importantly, however, we still clearly see, on average, forward-biased moves of KIF1A with the cargo.

### The stepping statics of KIF1A with a longer neck-linker

In this paper, we primarily focused on the specified molecular construct (C351) used in in-vitro motility assay experiments [19], [20], [21], in which the length of neck-linker except for His-tag is 22-residues. Here, to test robustness of our results, we investigated the stepping statics of another construct that has 5-residue longer neck-linker. The 5-residue segment is modeled as a flexible chain (by Modeller). In a similar way to the above sub-section, we estimated the range of the cargo position (Fig. S7B upper panel which shows the distribution of the probability density for the position of the cargo), and repeated simulations (10 runs each) with the initial cargo position at z = 6.25, 5.75, 5.25, 4.75, 4.25, 3.75, 3.25, 2.75, 2.25, 1.75, and 1.25 nm. From the initial position of the cargo: z>4.5 nm, we found clear forward-biased moves, whereas z<4.5 nm, the head seldom detached from MT (Fig. S7B lower panel). Thus, although the bias is weakened, we still see clear forward-biased moves of KIF1A with the cargo linked by 5-residue longer linker.

## Discussion

Conventional kinesin is dimeric and “walks” in a hand-over-hand fashion, akin to human walking by two legs. Extending the analogy to human walking, the current simulations suggest that the large cargo-analog can play the role of a cane for the walk of single-headed kinesin; with a cane, we can walk even with one leg.

Although in this work, we focused on a truncated KIF1A which is used in the single-molecular assay of Okada et al [19], [20], [21], we should note that the cellular function of KIF1A in vivo is markedly more complicated than the situation we considered here. Several experiments [40], [41] showed that KIF1A may be dimerized by virtue of being bound to a single cargo-analog in some case. Our model does not straightforwardly apply to the dimeric KIF1A system in vivo.

Next, we discuss in vitro experiments of related systems. First of all, forward-biased movements were observed for single-head kinesins, both KIF1A and a single-head construct of conventional kinesin mutant, with latex beads linked to C-terminus, where the size of beads are sub-µm to µm [13], [21]. Thus the current simulations are perfectly consistent with these results. In a study of myosin VI, single-molecule experiments reported very similar phenomenon to our simulations [38]. A single-head construct of myosin VI did not show directional movements without beads/cargo. When a bead was attached to an end of the myosin head, it exhibited directional movements. It was argued that the bead played the role of diffusion anchor.

Some other experiments in vitro are subtle. While a truncated single-headed kinesin (K351) did not show marked processive movements, it exhibited processive and directional movements when fused with BDTC (1.3-S subunit of propionibacterium shermanii transcarboxylase) [39]. Here, BDTC is much smaller than beads/vesicles and thus does not apparently correspond to the current simulations. Yet, the linked BDTC increased K351-BDTC affinity to MT which implies that BDTC attractively interacts with tubulin. Thus, the interaction of BDTC with MT may provide additional friction to the C-terminus of K351, which is qualitatively the same as the role of the cargo-analog in our simulations.

Next, we discuss the dynamics of KIF1A head and cargo-analog. In Video S2 and Fig. 4, the cargo looks almost fixed at the beginning. First, we note that, although we used the cargo-analog of ∼1 µm-size, Video. S2 and the snapshot in Fig. 4B drew much smaller ball to “visualize” the cargo position. Thus a small ball is purely for graphics. Taking into account the ∼1 µm-sized cargo together with the Stokes-Einstein law and Maxwell-Boltzmann distribution, we see that the cargo does not move much: Based on the Stokes-Einstein law *D*_KIF1A_ = 1.2×10^8^ [nm^2^/s], and *D*_cargo_ = 2.9×10^5^ [nm^2^/s], respectively, we estimate that, while the KIF1A head diffuses for ∼8 nm distance, the cargo diffuses only ∼0.4 nm which is rather small. Thus, it is physically reasonable that the cargo looks immobile in Video. S2, and Fig. 4. Furthermore, in Video S2 and Fig. 4A, the motions of KIF1A head look like Brownian dynamics with little effect of inertia, whereas the motions of the cargo-analog look under-damped oscillation. This difference can be understood by estimating the lifetime of the corresponding velocity correlations. We estimated that the lifetime of velocity correlation for the KIF1A head is ∼10 τ, and that for the cargo-analog is ∼10^8^ τ. A characteristic time scale of the detached KIF1A head to find the adjacent binding site (z = L or -L) from the dissociation was ∼10^6^ τ (see *Material and Methods*). Therefore, within this time scale, the motions of KIF1A should be diffusive, while the motion of the cargo-analog is damped oscillation.

Related to these arguments, we also note time scale for KIF1A head diffusion. As in Result, our estimate in simulations was τ_attachment_∼2.5 µs–6.4 µs. Experimentally, the mean duration time of weak-binding state was estimated as τ_w_ = 7.5 ms [19]. However, τ_w_ obtained via an indirect estimate seems to include not only the diffusional searching time, but also the ADP release time. Since ADP release is a slow process, we do not know much on the diffusional search time.

We next discuss the effect of neck-linker length on the enhanced forward-biased motions. After the neck-linker docking, the average positions of cargo for C351 and a 5-residue longer variant C351+5 were similar, while the distribution of the positions was broader for the C351+5 case than that for the C351 case. (Fig. S7 A upper panel). Based on Fig. S7, we can estimate that the average steps per one ATP cycle are about 2.7 nm for C351 and about 2.0 nm for C351+5. Thus, a longer neck linker contributes to broadening of the distribution of the cargo position at the time of ATP hydrolysis, which results is gradual decrease in the forward biased movement of KIF1A. The full-length KIF1A has much longer neck linker. In the recent simulation study for the dimeric kinesin with a long tail domain and cargo [42], the neck-linker docking itself did not bring the cargo to the forward position significantly. On top, the full length KIF1A tends to dimerize. So, our analysis focuses on the truncated KIF1A construct such as C351 and argument for the full-length system needs further analysis.

Importantly, the current simulations showed that the linked cargo-analog is *sufficient* to induce the biased Brownian movement of KIF1A, but whether the linked diffusion anchor is *necessary* or not was not investigated. In vitro experiments, KIF1A exhibited forward-biased Brownian movements even when only a chromophore was attached [19], [20], [21]. Since the chromophore is much smaller than the cargo/bead, this does not correspond to the current simulations. Note that His-tags attached to C-terminus of KIF1A in vitro constructs may also contribute to additional interactions with MT since tubulin contains negatively charged C-terminus tails (E-hooks) on the surface. The result that some constructs with too short neck-linker did not exhibit directional movements suggests importance of a certain length of neck-linker between the head and the His-tag. Yet, we do not exclude the possibility of other mechanisms that induce the directional movements. In particular, recent computational work reported that electrostatic interactions between two-head kinesin heads and MT can provide modest bias to the forward direction [22]. Thus, the linked cargo-analog can be to enhance the forward bias.

In the end, we briefly mention about possible experiments which can test the current simulation results. The direct test of the proposed mechanism is to perform a motility assay with a bead or dye attached to the core of the KIF1A head which is far from N- or C- termini and which is located on the surface opposite to the MT binding orientation. Even better way is to introduce two imaging probes; a bead in the C-terminus and a dye to the core of KIF1A head. We expect to see movements in asymmetric hand-over-hand fashion. Another easier but more indirect test is to investigate ion-strength dependence of the stepping statics for C351. If the interaction between KIF1A and MT is weakened by a higher ion-strength, the re-binding time of KIF1A becomes longer, which results in weakening of the forward-bias.

## Materials and Methods

### Simulated systems and structures

To simulate an ATP cycle by the structure-based CG model, we need reference KIF1A-MT complex structures X_ν_ for every states ν ( = T, D, Φ) in the cycle (T, D, and Φ correspond to ATP, ADP and nucleotide free state, respectively). The crystal structures of ATP-bound KIF1A (KIF1A(T)) and ADP-bound KIF1A (KIF1A(D)) are available from the Protein Data Bank and were used in the CG models as references. For the KIF1A-MT complex structures, we used the model structures 2HXF (the pdb id) for the KIF1A(T)/tubulin αβ complex X_T_, and 2HXH for the KIF1A(D)/tubulin αβ complex X_D_ [30], [31] (see Fig. 1A, 1B, and 1C). Since motility-assay experiments heavily used a chimera protein C351 where the catalytic core of KIF1A was fused to the neck linker of conventional kinesin (KIF5C) [19], [20], [21], we employed the same chimera C351 (KIF1A-KIF5C), except for N-terminal T7-tag and C-terminal His-tag and Cys. Missing residues in the loop of KIF1A, including a part of the neck-linker region, were modeled by Modeller [43]: We constructed 200-samples and chose the model with the best Modeller score. These modeled loops were treated as flexible regions with reduced force constants (see Coarse-grained model in Supporting Information Text S1).

For the nucleotide free state, no structure is available. Since KIF1A (Φ) constitutes the strong-binding state in a similar way to KIF1A (T), we decided to use the same structure as the X_T_ excluding the neck linker, which is known to be disordered in Φ-state [28], [44], [45], [46]. We treated the neck liner in Φ-state as flexible regions.

Both 2HXF and 2HXH contain missing residues in tubulin αβ at the so-called E-hook region, which were not explicitly modeled. Instead, we included the effect of E-hook in a simpler way (see Coarse-grained model in Supporting Information Text S1).

The simulation system here contains a KIF1A molecule and three copies of tubulin αβ dimers (Fig. 1D) where all the tubulin molecules were fixed throughout simulations. The initial structure of simulations contained X_T_ structure of KIF1A attached to the central tubulin αβ dimer at the form of 2HXF. The coordinates were set so that the MT protofilament is along the *z*-axis with the plus end being positive *z*, and KIF1A-binding surface of tubulin is roughly perpendicular to *y*-axis (see Fig. 1D). The origin was defined by the position of Cα-atom of Phe94 of KIF1A at the initial structure (roughly the center of mass of KIF1A). The period of the MT is about 8 nm along *z*-axis.

### Coarse-grained model

We applied the structure-based CG models for the KIF1A-MT system [34], [35]. KIF1A and three tubulin αβ dimers were represented by a set of beads, where each bead placed at the position of Cα atom represents one amino acid. (See Supporting Information Text S1 for detail).

### Simulation protocol and dynamics

To mimic an ATP hydrolysis cycle (Fig. 2A), we employed a simulation protocol summarized in Fig. 2B. The dynamics of the KIF1A protein were simulated by the underdamped Langevin equation at a constant temperature T = 290.0 K with CafeMol [47]. The step size *dt* of the time integration is *dt* = 0.1 τ, where τ∼0.128 (ps) is the unit of time in CG-simulation.
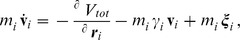
where ***v*** *_i_* is the velocity of the *i-*th bead and a dot represents the derivative with respect to time: *t* (thus, ***v*** *_i_* = 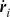. ***ξ*** *_i_* is a Gaussian white random force, which satisfies <***ξ*** *_i_*> = 0 and <***ξ*** *_i_*(*t*) ***ξ*** *_j_*(*t*′)> = 2m*_i_* γ*_i_* k_B_T δ*_ij_* δ(*t* -*t*′) **1**, where the bracket denotes the ensemble average and **1** is a 3
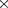
3 unit matrix. k_B_, is the Boltzmann constant, γ*_i_* and m*_i_* are the friction coefficient and the mass for the one residue. (See Supporting Information Text S1 for more detail).

### The units and time scales of CG-simulations

The units of CG-simulations are given as follows: The length unit is 0.1 nm. The energy unit is kcal/mol (∼6.95 pN.nm). The unit of mass can be defined as setting m*_i_*, the mass for an amino acid. We set m*_i_* = 10, which is just a convention in CafeMol, which leads to the mass unit as 2.275
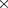
10^−26^ kg.

The friction coefficient γ*_i_* for a residue is decided so that the diffusion constant of the KIF1A head in simulations roughly agrees with that (1.2×10^8^ [nm^2^/s]) by the Stokes-Einstein law: By setting γ*_i_* = 0.1 for an amino acid, we obtained a reasonable diffusion coefficient of KIF1A head (*D*_KIF1A_ = 1.58×10^−5^ [nm^2^/τ]) in simulation and the time unit in CG-simulation τ ∼0.128 (ps).

As for the default-sized cargo, we modeled its radius 3000 times as large as the radius of an amino acid, which is ∼1 µm. So, the mass m_cargo_ of the cargo scales as m*_i_*×3000^3^, which gives 2.7×10^11^. The friction coefficient γ_cargo_ for the cargo is 0.1×3000/3000^3^ = 1.1×10^−8^ [1/τ].

In this paper, we used the underdamped Langevin dynamics as the equation of motions. We note that, even when we use the underdamped Langevin equation, it gives us overdamped motions when we investigate motions in time scales longer than the velocity correlation time. For an amino acid, the velocity correlation is given by 1/γ_i_ = 10 τ (where the simulation time unit τ corresponds to ∼0.128 ps in real time scale). For the KIF1A head, we computed the lifetime of the velocity correlation of the center of mass, which was ∼10 τ, while that for the cargo was ∼10^8^ τ. Thus, for the characteristic time scale t_c_ ∼L^2^/2*D*_KIF1A_ ∼10^6^ τ of the KIF1A head to find the adjacent binding site (z = L or -L) from the dissociation, an amino acid and the KIF1A head behave as overdamped, while the cargo motion is underdamped.

## Supporting Information

Figure S1 **Position distribution of KIF1A-head and C-terminus in one ATP-hydrolysis cycle with the electrostatic interaction [stand-alone/weak/DH].** This figure shows the position distributions of KIF1A-head (blue) and C-terminus (orange) for [stand-alone/weak/DH] case. One ATP cycle was split into 6-phases. In the top panel, the vertical dashed lines show the initial positions. In the bottom panel, the red-letter value indicates the P-value to observe the current data based on the null hypothesis (see Table 1 for more detail).
(TIF)

Figure S2 **Translational movements of stand-alone KIF1A with the electrostatic interaction between KIF1A and MT [stand-alone/weak/DH].** This figure shows movements for the case of a weak go interaction (ε_go_^KIF1A-MT^ = 0.153) between KIF1A and MT with the electrostatic interaction denoted as [stand-alone/weak/DH]. The blue and orange lines are z-coordinates of the KIF1A-head (the center of mass) and C-terminal, respectively. Each trajectory contains 4 phases, T, D, Φ, and the next T states split by dashed lines. Note that the scale in x-axis changes at 5×10^5^ τ, where τ∼0.128 ps is the unit of time in CG simulations. We confirm that the qualitative tendency of the sample trajectory for the coordinate is very similar to the system [stand-alone/weak].
(TIF)

Figure S3 **Representative time courses of dissociation of the head for system with the electrostatic interaction [stand-alone/weak/DH].** (A) The probability of the KIF1A head still attached on (not yet dissociated from) MT as a function of duration time from the T → D switch, i.e., time after V_MB_ (R|X_T_, X_D_, ΔV_1_) → V_MB_ (R|X_T_, X_D_, ΔV_2_) switch. The gray line corresponds to the case of the system with electrostatic interaction [stand-alone/weak/DH]. The color assignments for the others lines are same as Figure 6 (A). Interestingly, the electrostatic interaction enhances both the detachment and the attachment ratios compared with the case [stand-alone/weak]. (B) The binding energy between the KIF1A head and MT as a function of duration time from the T → D switch for case of the case of the weak interaction with electrostatic interaction [stand-alone/weak/DH]. The blue line corresponds to the Go interaction between KIF1A and MT, while the red line corresponds to the electrostatic interaction between KIF1A and MT for the system [stand-alone/weak/DH]. We see that the electrostatic energy and Go-like interaction change in the opposite way; the native interface has electrostatic frustration. The electrostatic energy is, although not negligible, somewhat weaker than that of Go-like interaction.
(TIF)

Figure S4 **The attachment and the diffusion processes in D state for system with electrostatic interaction [stand-alone/weak/DH].** (A) The gray line shows the attachment probabilities as a function of the duration time after the dissociation of the KIF1A head from MT for the cases of [stand-alone/weak/DH]. The other lines are also depicted just for the comparison. The color assignments for the others lines are same as Figure 7 (A). (B) The statistics of the head and the cargo-analog positions at 1×10^6^ τ and 5×10^6^τ after the dissociation of the head from MT in D-state. The meanings of the blue and orange bar are the same as those in Figure 5.
(TIF)

Figure S5 **The C-terminus displacement upon neck-linker docking in T-state for the system with electrostatic interaction [stand-alone/weak/DH].** The time evolutions of C-terminus as a function of time for the cases of [stand-alone/weak/DH]. The time zero corresponds to the time of the switch from Φ to T state corresponding to the ATP binding. The statistics includes only samples that landed at the 8-nm forward binding site.
(TIF)

Figure S6 **The statics of the re-dissociation and the reattachment process for system with electrostatic interaction [stand-alone/weak/DH].** The upper panel shows the re-dissociation probability at each-binding site, while the lower one indicates the reattachment probability of the head. The state that caused the re-dissociation/reattachment event was shown by different colors; the red (D-state), blue (T-state), and cyan (Φ-state). T he re-dissociation and re-attachment process in the case [standalone/weak/DH] do not induce the significantly forward biased movement.
(TIF)

Figure S7 **Stepping statics with various initial position of cargo for system with default and longer neck-linker.** (A) The upper panel: the probability density for the relative position of the cargo at the end of one ATP-cycle (after Neck-linker docking phase) for the case with default truncated neck-linker (C351). The lower panel: the stepping statics of the one-ATP cycle simulation with various initial condition, where we conducted 10-sample simulations for each initial cargo position (z = 4.75, 4.25, 3.75, 3.25, 2.75, 2.25, 1.75, and 1.25 nm), respectively. The blue, green, yellow, and red bars in the histogram stands for the frequency of the forward-stepping, 0 nm-stepping, backward-stepping, and no-dissociation of the head from MT, respectively. (B) The upper panel: the probability density for the relative position of the cargo at the end of one ATP-cycle for a 5-residues longer neck-linker. (The extended 5-residues of the neck-linker was modeled by Modeller.) The lower panel: the stepping statics of the additional one-ATP cycle simulation with various initial condition, where we conducted 10-sample simulations for each initial cargo position (z = 6.25, 5.75, 5.25, 4.75, 4.25, 3.75, 3.25, 2.75, 2.25, 1.75, and 1.25 nm), respectively. The blue, green, yellow, and red bars in the histogram stand for the frequency of the forward-stepping, 0 nm-stepping, backward-stepping, and no-dissociation of the head from MT, respectively.
(TIF)

Text S1 **The detail information for** **Materials and Methods** **.** The detail information for Coarse-grained model and Simulation protocol and dynamics.
(DOC)

Video S1 **An example of stand-alone KIF1A movements for [stand-alone/weak].** Starting from the initial structure (Fig. 1 D), KIF1A took one ATP cycle (X_T_→X_D_→X_Φ_→X_T_), and finally returned to the original site (0 nm).
(MPG)

Video S2 **An example of movements of KIF1A with a cargo for [cargo/strong].** Starting from the initial structure (Fig. 1 D), KIF1A with a cargo-analog took one ATP cycle (X_T_→X_D_→X_Φ_→X_T_), and exhibited an 8-nm forward step. The simulation treated the default-cargo as ∼1 µm-sized sphere, but this video used much smaller ball to “visualize” the cargo position, purely for graphics.
(MPG)
