# Supplementary figures and images for: Structure-based Molecular Simulations Reveal the Enhancement of Biased Brownian Motions in Single-headed Kinesin
Source: PLoS Comput Biol. 2013 Feb 14;9(2):e1002907. doi: 10.1371/journal.pcbi.1002907 (PMC3572960; doi:10.1371/journal.pcbi.1002907)

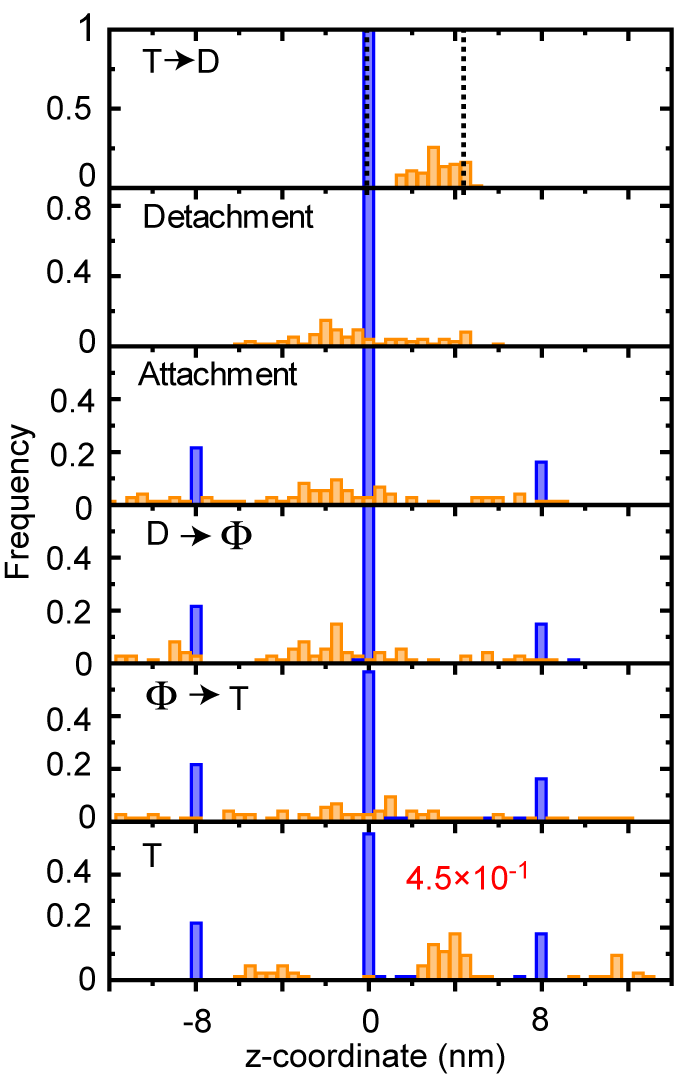

Supplement: Figure S1 — Position distribution of KIF1A-head and C-terminus in one ATP-hydrolysis cycle with the electrostatic interaction [stand-alone/weak/DH]. This figure shows the position distributions of KIF1A-head (blue) and C-terminus (orange) for [stand-alone/weak/DH] case. One ATP cycle was split into 6-phases. In the top panel, the vertical dashed lines show the initial positions. In the bottom panel, the red-letter value indicates the P-value to observe the current data based on the null hypothesis (see Table 1 for more detail). (TIF) [file pcbi.1002907.s001.tif]

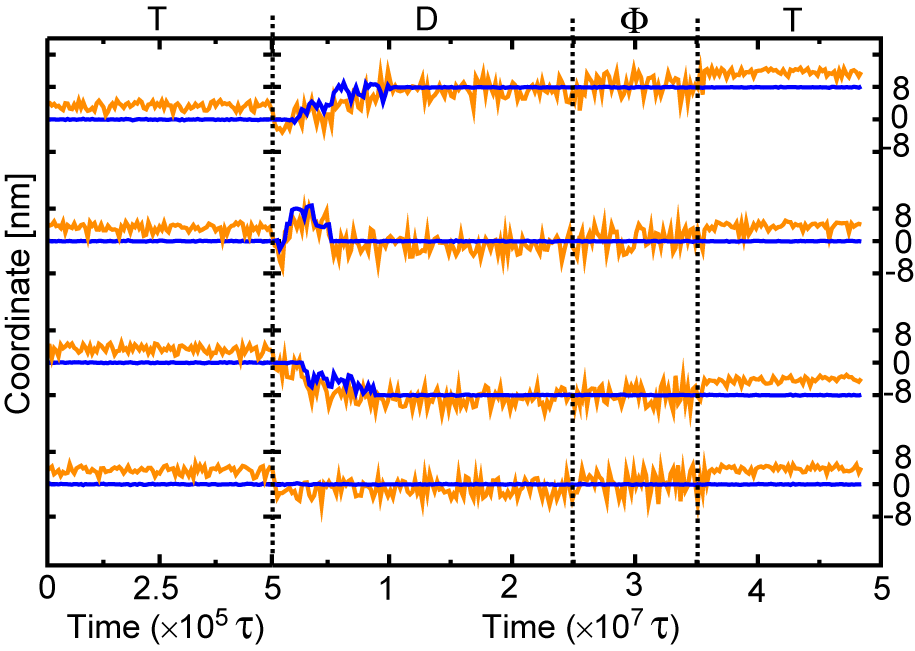

Supplement: Figure S2 — Translational movements of stand-alone KIF1A with the electrostatic interaction between KIF1A and MT [stand-alone/weak/DH]. This figure shows movements for the case of a weak go interaction (εgo KIF1A-MT = 0.153) between KIF1A and MT with the electrostatic interaction denoted as [stand-alone/weak/DH]. The blue and orange lines are z-coordinates of the KIF1A-head (the center of mass) and C-terminal, respectively. Each trajectory contains 4 phases, T, D, Φ, and the next T states split by dashed lines. Note that the scale in x-axis changes at 5×10^5^ τ, where τ∼0.128 ps is the unit of time in CG simulations. We confirm that the qualitative tendency of the sample trajectory for the coordinate is very similar to the system [stand-alone/weak]. (TIF) [file pcbi.1002907.s002.tif]

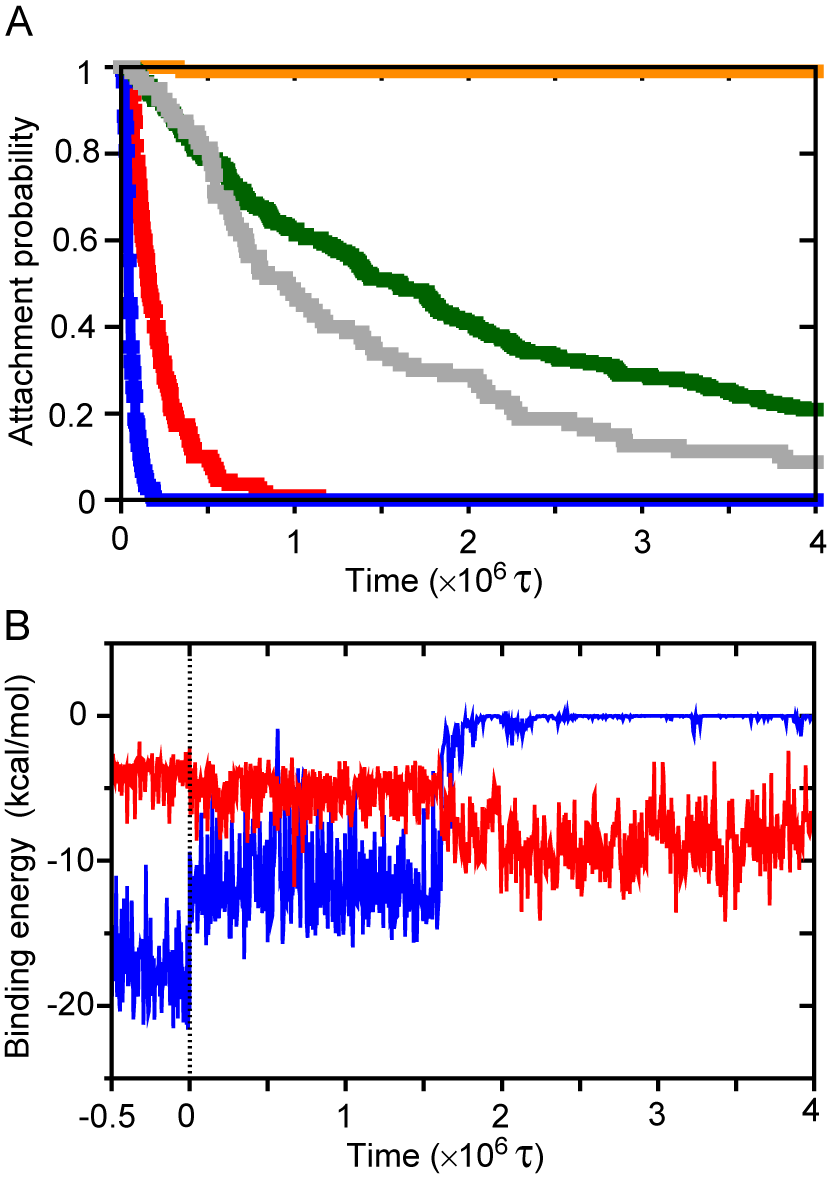

Supplement: Figure S3 — Representative time courses of dissociation of the head for system with the electrostatic interaction [stand-alone/weak/DH]. (A) The probability of the KIF1A head still attached on (not yet dissociated from) MT as a function of duration time from the T → D switch, i.e., time after VMB (R|XT, XD, ΔV1) → VMB (R|XT, XD, ΔV2) switch. The gray line corresponds to the case of the system with electrostatic interaction [stand-alone/weak/DH]. The color assignments for the others lines are same as Figure 6 (A). Interestingly, the electrostatic interaction enhances both the detachment and the attachment ratios compared with the case [stand-alone/weak]. (B) The binding energy between the KIF1A head and MT as a function of duration time from the T → D switch for case of the case of the weak interaction with electrostatic interaction [stand-alone/weak/DH]. The blue line corresponds to the Go interaction between KIF1A and MT, while the red line corresponds to the electrostatic interaction between KIF1A and MT for the system [stand-alone/weak/DH]. We see that the electrostatic energy and Go-like interaction change in the opposite way; the native interface has electrostatic frustration. The electrostatic energy is, although not negligible, somewhat weaker than that of Go-like interaction. (TIF) [file pcbi.1002907.s003.tif]

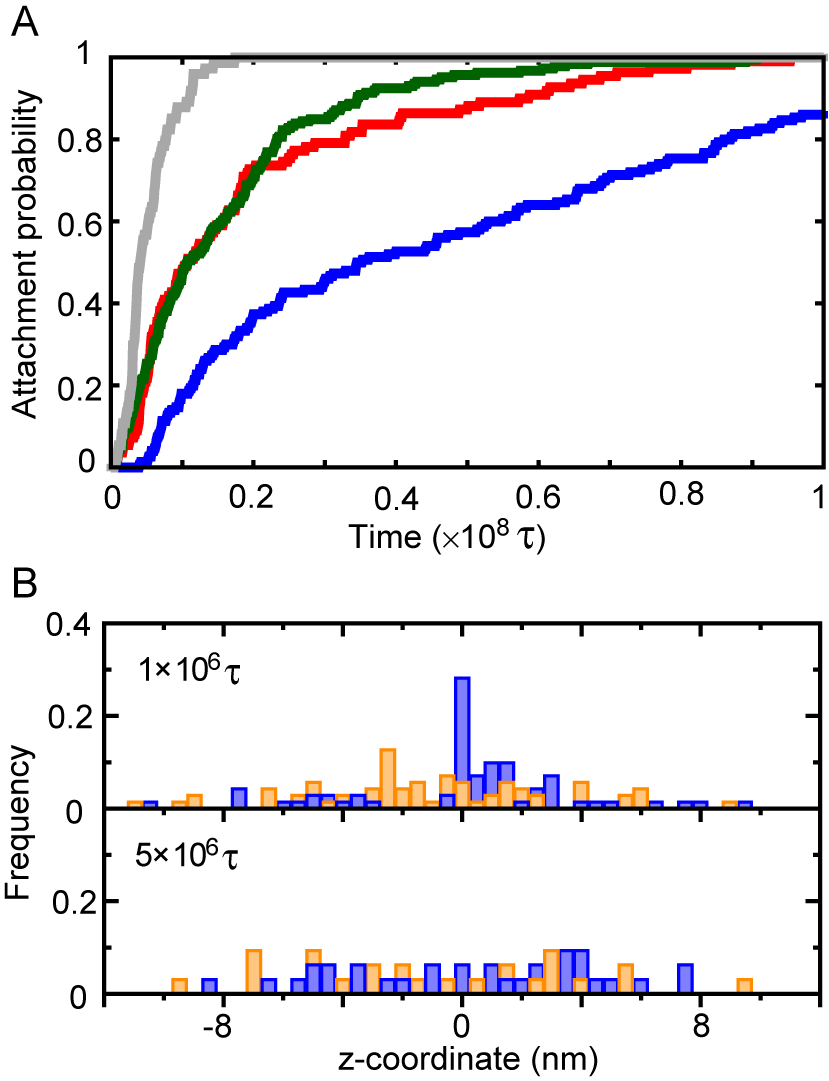

Supplement: Figure S4 — The attachment and the diffusion processes in D state for system with electrostatic interaction [stand-alone/weak/DH]. (A) The gray line shows the attachment probabilities as a function of the duration time after the dissociation of the KIF1A head from MT for the cases of [stand-alone/weak/DH]. The other lines are also depicted just for the comparison. The color assignments for the others lines are same as Figure 7 (A). (B) The statistics of the head and the cargo-analog positions at 1×10^6^ τ and 5×10^6^τ after the dissociation of the head from MT in D-state. The meanings of the blue and orange bar are the same as those in Figure 5. (TIF) [file pcbi.1002907.s004.tif]

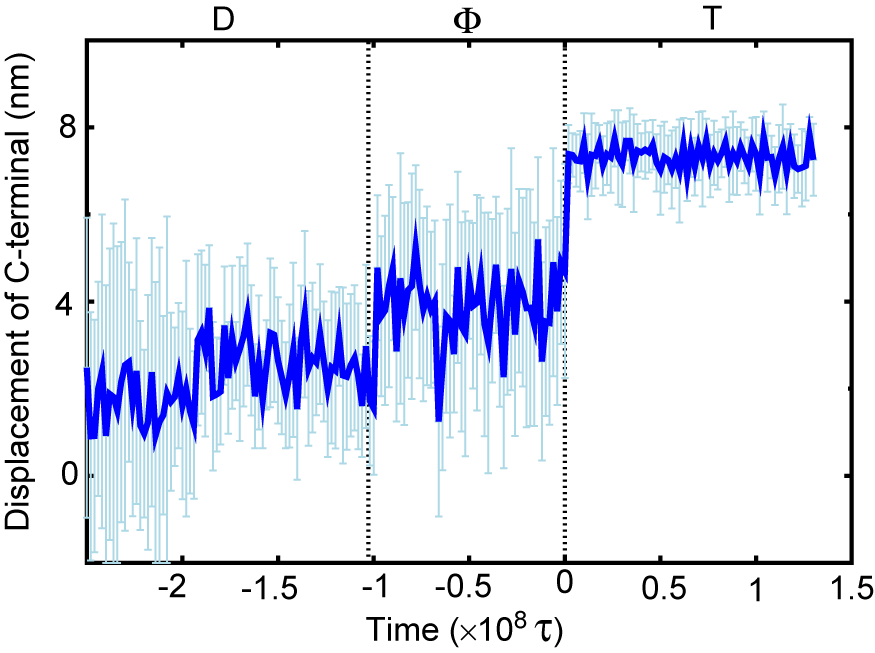

Supplement: Figure S5 — The C-terminus displacement upon neck-linker docking in T-state for the system with electrostatic interaction [stand-alone/weak/DH]. The time evolutions of C-terminus as a function of time for the cases of [stand-alone/weak/DH]. The time zero corresponds to the time of the switch from Φ to T state corresponding to the ATP binding. The statistics includes only samples that landed at the 8-nm forward binding site. (TIF) [file pcbi.1002907.s005.tif]

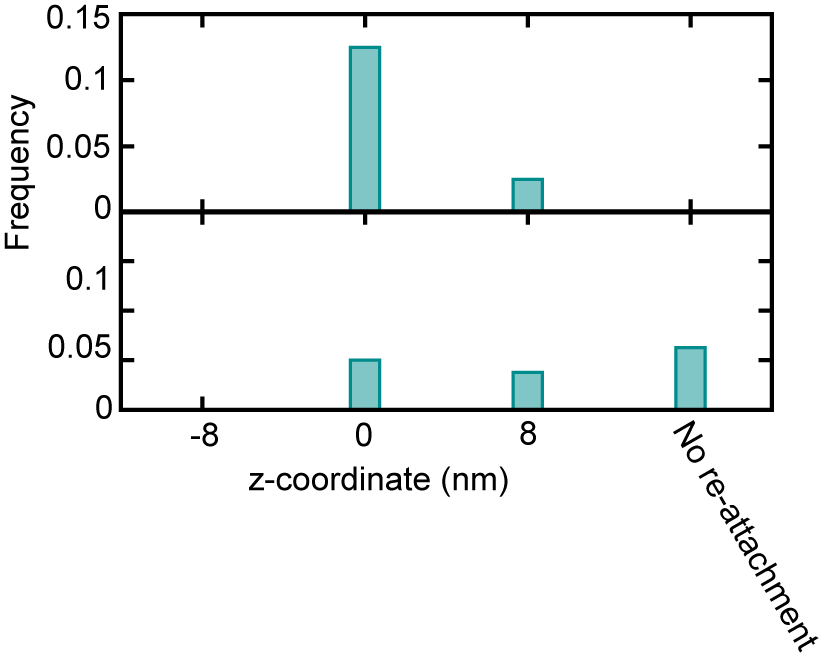

Supplement: Figure S6 — The statics of the re-dissociation and the reattachment process for system with electrostatic interaction [stand-alone/weak/DH]. The upper panel shows the re-dissociation probability at each-binding site, while the lower one indicates the reattachment probability of the head. The state that caused the re-dissociation/reattachment event was shown by different colors; the red (D-state), blue (T-state), and cyan (Φ-state). T he re-dissociation and re-attachment process in the case [standalone/weak/DH] do not induce the significantly forward biased movement. (TIF) [file pcbi.1002907.s006.tif]

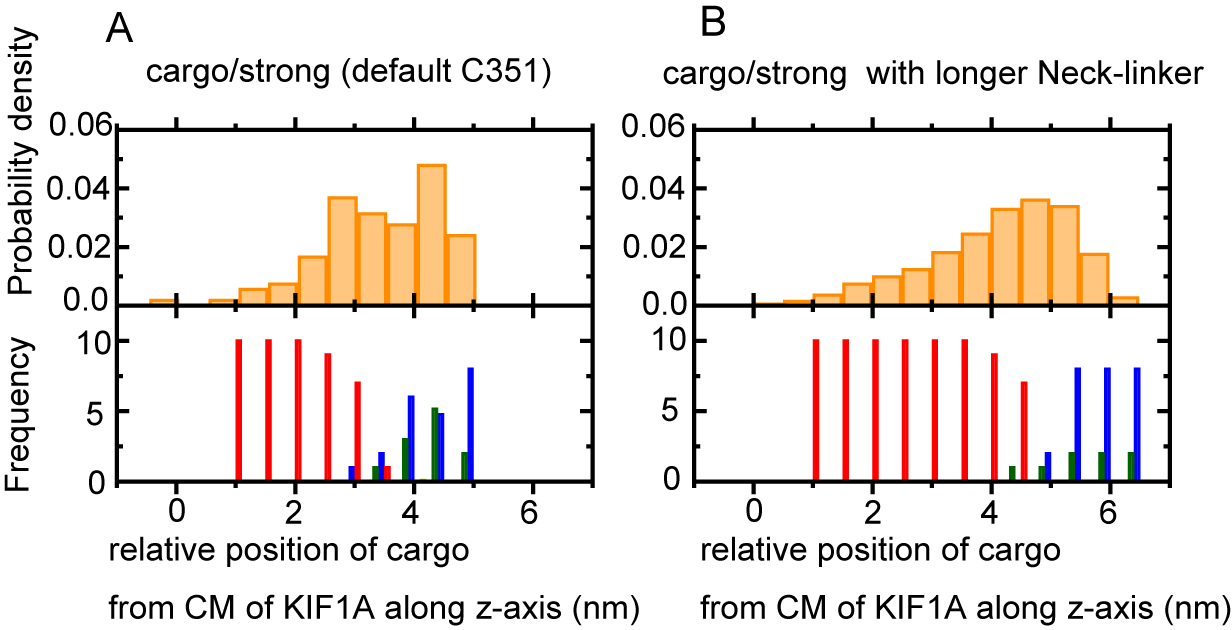

Supplement: Figure S7 — Stepping statics with various initial position of cargo for system with default and longer neck-linker. (A) The upper panel: the probability density for the relative position of the cargo at the end of one ATP-cycle (after Neck-linker docking phase) for the case with default truncated neck-linker (C351). The lower panel: the stepping statics of the one-ATP cycle simulation with various initial condition, where we conducted 10-sample simulations for each initial cargo position (z = 4.75, 4.25, 3.75, 3.25, 2.75, 2.25, 1.75, and 1.25 nm), respectively. The blue, green, yellow, and red bars in the histogram stands for the frequency of the forward-stepping, 0 nm-stepping, backward-stepping, and no-dissociation of the head from MT, respectively. (B) The upper panel: the probability density for the relative position of the cargo at the end of one ATP-cycle for a 5-residues longer neck-linker. (The extended 5-residues of the neck-linker was modeled by Modeller.) The lower panel: the stepping statics of the additional one-ATP cycle simulation with various initial condition, where we conducted 10-sample simulations for each initial cargo position (z = 6.25, 5.75, 5.25, 4.75, 4.25, 3.75, 3.25, 2.75, 2.25, 1.75, and 1.25 nm), respectively. The blue, green, yellow, and red bars in the histogram stand for the frequency of the forward-stepping, 0 nm-stepping, backward-stepping, and no-dissociation of the head from MT, respectively. (TIF) [file pcbi.1002907.s007.tif]
