# Supplementary material for: Structure-based Molecular Simulations Reveal the Enhancement of Biased Brownian Motions in Single-headed Kinesin
Source: PLoS Comput Biol. 2013 Feb 14;9(2):e1002907. doi: 10.1371/journal.pcbi.1002907 (PMC3572960; doi:10.1371/journal.pcbi.1002907)
Supplement: Text S1 — The detail information for Materials and Methods . The detail information for Coarse-grained model and Simulation protocol and dynamics. (DOC) [file pcbi.1002907.s008.doc]

**Structure-based molecular simulations reveal the enhancement of biased Brownian motions in single-headed kinesin:**

**Text S1 (Supporting Information)**

Ryo Kanada,* Takeshi Kuwata,^†^ Hiroo Kenzaki,* and Shoji Takada*^‡^

**Department of Biophysics Graduate School of Science, Kyoto University, Kyoto 606-8502, Japan; ^†^Graduate School of Science and Technology, Kobe University, Kobe 657-8501, Japan; ^‡^CREST Japan Science and Technology Agency, Kawaguchi, Saitama 332-0012, Japan*

Correspondence author

Shoji Takada

Department of Biophysics, Graduate School of Science, Kyoto University, Kyoto 606-8502, Japan

Phone: 81-75-753-4220, Email: takada@biophys.kyoto-u.ac.jp

**Materials and Methods**

**Coarse-grained model**

We applied the structure-based CG models for the KIF1A-MT system . KIF1A and three tubulin αβ dimers were represented by a set of beads, where each bead placed at the position of Cα atom represents one amino acid. The total potential energy function is defined as

where *V*^KIF1A^ is the energy for intra-chain interaction of KIF1A, *V*^KIF1A-MT^ is the inter-chain interaction between KIF1A and tubulin αβ, *U*^tarzan^ represents the loose electrostatic attraction between K-loop and E-hooks in a simplified form, and *U*^exvol(MT)^ is to simply represent an excluded volume effect from other protofilaments of MT (note that MT is composed of 13 protofilaments, while the simulated system contained only one protofilament).

During an ATP hydrolysis cycle, KIF1A changes its chemical state, which is represented here by dynamically switching the potentials, *V*^KIF1A^ + *V*^KIF1A-MT^ following the simulation scheme summarized in Fig. 2B. The potential *V*^KIF1A^ takes either a single-basin potential, or a double basin potential. Below, we briefly summarize them because the complete formula have been described in detail.

The single-basin potential *V*(R| X_ν_) (ν= T, D, Φ) (X_ν_ denotes the reference structure) is the off-lattice Go-like model proposed by Clementi et al.. The potential for the intra-chain part and the inter-chain part are defined by

where **R** is the Cartesian coordinates of the simulated protein, b*_i_* is the *i*-th virtual bond length defined as |***r*** *_i+1_* – ***r*** *_i_* |, where ***r*** *_i_* stands for the Cartesian coordinate of the *i*-th amino acid, θ*_i_* is the *i*-th bond angle between two consecutive virtual bond vectors, ***r****_i+1_*-***r****_i_*, and ***r****_i+2_*-***r****_i+1_*, φ*_i_* is the *i*-th dihedral angle around the *i+1*-th virtual bond ***r****_i+2_*-***r****_i+1_*, and r*_ij_* is the distance between *i*-th and *j*-th amino acids. All parameters with the subscript ν are the constants which have the values of the corresponding variables at the reference structure X_ν_. For the parameters of the intra-chain interaction, we set the following way: K_b_ = 100.0, K_θ_= 20.0, Kφ^(1)^ =1.0, Kφ^(3)^ =0.5, ε_go_^KIF1A^=0.36, ε_ev_=0.2, *d*=4Ǻ, except for the disordered loop regions (see below). (Throughout the paper, the energy unit corresponds to kcal/mol (~ 1.7 k_B_ T = ~6.95 pN.nm) although the mapping is rather approximate). For the strength of the inter-chain interaction we mainly investigated the following two cases in this article. The parameter for the strong interaction case is ε_go_^KIF1A-MT^ =0.225, while one for the weak interaction case is ε_go_^KIF1A-MT^=0.153. For the disordered and modeled loop, we reduced K_θ_, Kφ^(1)^, Kφ^(3)^, ε_go_^KIF1A^, ε_go_^KIF1A-MT^ by a factor of 0.01. The summation is over the native contact pairs, pairs of amino acids that are physically close to each other at the reference structure: If one of the non-hydrogen atoms in the *i*-th amino acid is within 6.5 Ǻ from any non-hydrogen atom in the *j*-th amino acid, we define the pair of the *i*-th and the *j*-th amino acids as being the native contact.

The double-basin potential realizes an energy landscape that is globally funnel-shaped and that has two basins at the bottom of the funnel. It relies on the availability of two reference structures X_α_, and X_β_. The potential is defined as

where V_Go'_(R| X_ν_) is essentially the Clementi's off-lattice Go potential V(R| X_ν_) but is slightly modified by technical reasons. The double basin potential contains the two additional parameters: The relative stability Δ*V* of two-basins and the coupling constant Δ that modulates the energy barrier height between the two basins. We set Δ =100 throughout the work.

The value of Δ*V* depends on the phase. As in Fig. 2B, Δ*V*_1_ should be positive to make X_T_ the stable basin, while Δ*V*_2_ should be negative to make X_D_ the stable basin. Similarly, Δ*V*_3_ is positive, and Δ*V*_4_ is negative. Specifically, we chose Δ*V*_1_=200, Δ*V*_2_= - 100, Δ*V*_3_ = 100, Δ*V*_4_= - 200, where qualitative results were not sensitively affected by these values.

As noted before, *U*^tarzan^ represents the loose electrostatic attraction between K-loop of KIF1A and E-hooks of tubulins in a simplified form. We added the constraint potential using the coordinates of Lys297 (***r***_K-loop_ ) and the coordinates of Ser439 in α-tubulin and of Asp437 in β-tubulin (***r***_E-hook(_*_j_*) (1*j*7) where *j* is numbered from minus end (*j*= 1) to plus end of MT (*j*= 6) in the actual simulation system and *j* = 7 corresponds to the α-tubulin monomer which is virtually added to the system (The all positions of the ***r***_E-hook(_*_j_*) are depicted as red sphere in Fig. 1D).

where the strength parameter *C*_tarzan_ = 10, and the characteristic length *r*_tarzan_= 55.0Ǻ were used. We explain the reason why ***r***_E-hook(_*_7_*) should be considered explicitly. ***r***_E-hook(_*_j_*) locates around the left side edge of each tubulin monomer (red dots in Fig. 1D), and thus, without including ***r***_E-hook(_*_7_*), asymmetric *U*^tarzan^ can obviously generate the backward bias.

Our simulation system contains a single protofilament of MT, whereas the real MT is made of 13-protofilaments. (It has been shown that kinesin proceeds along a single protofilament) To take into account geometry of other protofilaments, we added a penalty term for KIF1A to invade the inner side of MT-filament, which is defined as y_MT_(x): y < y_MT_(x), where x, y, and z-axis are shown in Fig. 1D. Specifically, we applied a repulsive interaction to the coordinate of Phe94 of KIF1A, which is near the center of the mass of KIF1A, defined as

where y_CM_ is the y-coordinate for Phe94, *C*_MT_ =1, *r*_dist_=4.0 Ǻ, and *y*_MT_=-45.95 Ǻ which corresponds to the red dashed line in Fig. 1D.

**Simulation protocol and dynamics**

To mimic an ATP hydrolysis cycle (Fig. 2A), we employed a simulation protocol summarized in Fig. 2B. Here, we describe it in more detail. (i) As the initial structure at time step=0, we set up the structure depicted in Fig. 1D, where KIF1A(T) is attached to the central tubulin dimer (z=0). With V_MB_(R| X_T_, X_D_, ΔV_1_), we simulated the system 510^5^ τ, where τ (~0.128 ps) is the unit of time in CG-simulation as mentioned in the *Materials and Methods* in the main-text. (ii) After that, we switched the potential to V_MB_(R| X_T_, X_D_, ΔV_2_) corresponding to the ATP hydrolysis and Pi release, and simulated the system for 410^6^ τ. (iii)Then, we changed the potential to the single basin one V(R|X_D_) (This is merely to save computer time), and simulated it for τ_D_ until KIF1A binds to MT. The duration τ_D_ depends on the time (τ_attach_) of the KIF1A head attachment on MT. After KIF1A attachment on MT, we continued simulations at least 1x10^7^ τ for relaxation. (iv) After the τ_D_, we changed the potential to V_MB_(R| X_Φ_, X_D_, ΔV_3_), and simulated it for 510^5^ τ. (v) After that, to induce a conformational change, we switched the potential to V_MB_(R| X_Φ_, X_D_, ΔV_4_) corresponding to ADP release, and simulated for 510^5^ τ. (vi) Then, we changed the potential to the single basin potential V(R|X_Φ_) (This is to save computer time, again), and simulated it for 110^7^ τ. (vii) We then changed the potential to V(R| X_T_) that corresponds to ATP binding, and simulated it for τ_T_ (τ_T_ <2 10^8^ τ).

The dynamics of the KIF1A protein were simulated by the underdamped Langevin equation at a constant temperature T= 290.0 K.

where ***v****_i_* is the velocity of the *i-*th bead and a dot represents the derivative with respect to time:

*t* (thus, ***v****_i_* =. ***ξ****_i_* is a Gaussian white random force, which satisfies <***ξ****_i_* > = 0 and <***ξ****_i_*(*t*) ***ξ****_j_*(*t*')>=2m*_i_* γ*_i_* k_B_T δ*_ij_* δ(*t* -*t*') **1**, where the bracket denotes the ensemble average and **1** is a 3 3 unit matrix. k_B_, is the Boltzmann constant. The step size *dt* of the numerical time integration is *dt* = 0.1τ = 1.28 ×10^-14^ [s], where τ (~0.128 ps) is the unit of time in CG-simulation. The friction coefficient γ*_i_* = 0.1 and mass m*_i_*=10.0 for the one residue as mentioned in *Materials and Methods* in the main-text. The friction coefficient and mass for the default-size cargo of which radius (~1 μm) is 3000-times as large as one-Cα bead are 1.1 10^-8^ and 2.7 10^11^, respectively. (Provided that the density for cargo is same as that for Cα-bead, the mass for cargo of which radius is 3000-times as large as radius of Cα-bead is estimated as 103000^3^ =2.7 10^11^ [CG-mass unit]. If the stokes-law is satisfied, since the ratio of (m*_i_* γ*_i_*) between cargo and Cα bead should be proportional to the ratio of radius between cargo and Cα bead, the friction coefficient γ*_i_* for the default cargo can be estimated as γ*_i_* = 0.1/ 3000^2^ =1.1110^-8^ [1/τ].) Here, the friction and mass are in the unit of CafeMol (CG) (<http://www.cafemol.org/>). Throughout the simulations, tubulins were fixed.
